# Dissecting Yield Architecture and Trait Interactions in Rice Using Integrative Multivariate Selection Index and Phenotypic Similarity Analysis

**DOI:** 10.3390/plants15142134

**Published:** 2026-07-10

**Authors:** Chandrasekhar Manikala, Rupeshwar Naik Chinna, Thanet Khomphet

**Affiliations:** 1 Department of Genetics and Plant Breeding, Naini Agriculture Institute, Sam Higginbottom University of Agriculture, Technology and Sciences, Prayagraj 211007, Uttar Pradesh, India; chandrasekharmanikala003@gmail.com (C.M.); rupeshwar97@gmail.com (R.N.C.); 2 School of Agricultural Technology and Food Industry, Walailak University, Nakhon Si Thammarat 80160, Thailand; 3 Food Technology and Innovation Research Center of Excellence, Walailak University, Nakhon Si Thammarat 80160, Thailand

**Keywords:** rice (*Oryza sativa* L.), genetic variability, multi-trait selection, path analysis, principal component analysis, yield architecture

## Abstract

Rice yield is a complex polygenic trait influenced by intricate interactions among component characters. In this study, integrative quantitative genetics and multivariate models were used to dissect yield architecture and trait networks, and identify the promising genotypes among 21 rice genotypes that were tested in a randomized block design with three replicates. Analysis of variance revealed highly significant genotypic differences (*p* < 0.001) for most agronomic, yield, and grain-quality traits. Genetic variability analysis revealed high genotypic and phenotypic coefficients of variation, together with high heritability and genetic advance, for grain density per panicle, number of grains per panicle, biomass, flag leaf area, tillering ability, harvest index, and grain yield, indicating considerable genetic potential for genetic improvement. Pearson’s correlation and path coefficient analyses revealed that biomass and number of grains per panicle were the major determinants of grain yield per hill, with biomass exhibiting the strongest positive association (*r* = 0.678) and the largest direct effect. Principal component analysis indicated that the first two principal components explained 51.4% of the total phenotypic variation, with yield components, biomass, tillering traits, and grain-quality attributes contributing most strongly to genotype differentiation. Hierarchical cluster analysis grouped the genotypes into four distinct clusters, revealing substantial phenotypic divergence and valuable parental combinations for hybridization. The multi-trait selection index identified VAR16 as the most promising genotype, followed by VAR1, VAR17, VAR18, and VAR12, owing to their desirable combination of high grain yield and superior grain quality. Overall, this study offers a robust foundation for ideotype breeding and parental selection to enhance rice productivity and grain quality under subtropical conditions.

## 1. Introduction

Rice (*Oryza sativa* L.) is one of the most significant staple crops in the world, feeding more than half of the world’s population and contributing substantially to food security, especially in Asia and sub-Saharan Africa [1]. It contributes almost 20 percent of the world’s caloric intake and plays a central role in the livelihood of millions of smallholder farmers [2,3]. The crop is characterized by a high level of genetic diversity, with a broad spectrum of morphological, physiological, and grain-quality characteristics, which provides a valuable resource for breeding programs to increase the yield and adapt to various agro-ecological conditions [4,5]. Although there is astonishing improvement in breeding rice varieties during the Green Revolution, yield gains have plateaued in many regions due to genetic bottlenecks, environmental limitations, and the complex inheritance of yield and its related characteristics [6]. Grain yield in rice is a polygenic character that is controlled by various component characters, including the number of productive tillers, panicle length, number of grains per panicle, biomass, and harvest index. These attributes are often interrelated and influenced by both environmental and genetic factors, making the dissection of yield architecture not an easy task [7,8]. Understanding genetic variability and its interrelationships among these traits is therefore crucial for designing effective breeding strategies. Conventional selection based solely on yield can be inefficient because it has low heritability with high environmental impact. Consequently, breeders are increasingly employing indirect selection using yield-contributing traits that exhibit heritability and genetic gain [9].

Conventional studies of genetic variability of rice have primarily relied on univariate and bivariate statistical techniques, including analysis of variance, estimation of heritability, and correlation analysis, to offer valuable initial data, but these have been found limited in their ability to represent the complex, multidimensional relationships among traits related to yield [10,11]. Correlation analysis fails to distinguish between the direct and indirect effects, potentially leading to imprecise selection decisions when traits are interdependent. Moreover, numerous studies do not adequately explore the multivariate pattern of phenotypic diversity or determine the most important factors that cause variation among genotypes [12,13]. Furthermore, analytical tools such as PCA, path analysis, and clustering are usually used as isolated tools instead of within a cohesive framework. As a result, a gap persists in the formulation of integrative strategies that can explain trait relationships, causal effects, and the overall phenotypic structure to support more effective and efficient breeding approaches [12,14].

To address these limitations, the present study adopts an integrative quantitative genetic and multivariate approach to dissect yield architecture in rice. This framework integrates correlation analysis, path coefficient analysis, principal component analysis and phenotypic similarity analysis to give a comprehensive view of trait interactions and their contributions to yield. The correlation analysis is used as a preliminary measure to determine any significant association between traits, especially those associated with grain yield. However, to address these limitations, path coefficient analysis is applied to partition these correlations into direct and indirect effects, thereby revealing the true causal relationships among yield components [10,15]. This enables the identification of traits that exert a direct influence on yield and are therefore more reliable targets for selection.

Principal component analysis is utilized to reduce the dimensionality of the dataset and to identify the major components explaining the variation among genotypes. Analyzing the trait loadings on the principal components, one can define which traits are the most important in producing phenotypic variation and categorize genotypes with their multivariate patterns [14]. Also, phenotypic similarity analysis, which frequently relies on Euclidean distances and clustering algorithms, is employed to cluster genotypes into clusters with broadly comparable trait manifestations. This helps in identifying genetically different and complementary genotypes, which can be utilized in breeding programs to produce transgressive segregants and enhance yield potential [16]. By combining these analysis methods, a trait interaction network can be built to give a system-level view of yield formation. Such an approach not only enhances the accuracy of selection but also supports the development of ideotype breeding strategies by identifying key traits and their optimal combinations. Based on the above framework, the present study was undertaken with the following objectives: (1) To evaluate the extent of genetic variability among rice genotypes for yield and associated traits. (2) To determine the phenotypic and genotypic correlations among traits influencing grain yield. (3) To partition the direct and indirect effects of yield components using path coefficient analysis. (4) To identify the major contributors to phenotypic variation through principal component analysis. (5) To assess phenotypic similarity and classify genotypes into distinct clusters for identifying diverse parental lines.

## 2. Results

### 2.1. Phenotypic Variability and Coefficient of Variation of Rice Traits

#### 2.1.1. Variation in Vegetative Growth, Phenology, and Tillering Ability Among Rice Genotypes

**Figure 1:**
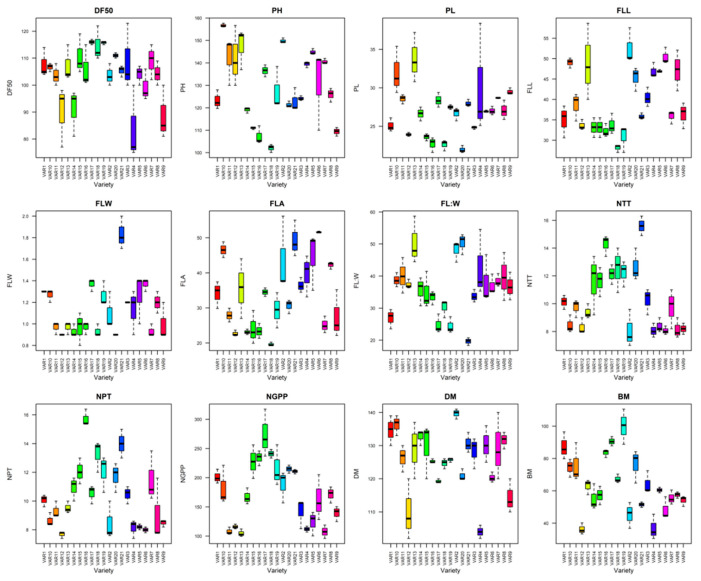
Distribution of key agro-morphological, phenological, and vegetative traits among 21 genotypes. Boxplots represent variation in traits, including (DF50, NTT, NPT, PH, PL, NGPP, BM, DM), leaf traits (FLA, FL:W, FLL, FLW). Boxes represent the interquartile range (IQR), the horizontal line within each box indicates the median, whiskers extend to 1.5 × IQR and represent outliers.

The mean comparison based on Duncan’s Multiple Range Test (DMRT) at the 5% significance level revealed significant phenotypic variation among the twenty-one rice genotypes for vegetative growth, phenological, tillering, biomass, and sink-related traits (Table 1). Genotypes were classified into multiple significance groups for each trait, reflecting broad genetic diversity in growth and developmental characteristics. The observed differences in mean performance, together with the distribution patterns illustrated by the boxplots (Figure 1), highlight the contrasting phenotypic expression of the evaluated germplasm and demonstrate their potential for selection in rice breeding programs.

Days to 50% flowering (DF50) ranged from 84.67 ± 4.04 days (VAR9) to 113.67 ± 2.08 days (VAR17), indicating the presence of both early- and late-maturing genotypes suitable for contrasting production systems. Likewise, days to maturity (DM) varied from 104.00 ± 1.00 days (VAR18) to 139.67 ± 1.53 days (VAR20), confirming substantial diversity in crop duration. The boxplots (Figure 1) clearly illustrate the separation between early- and late-maturing groups with only limited overlap, demonstrating the consistency of phenological differences across replications.

Plant height exhibited considerable variation, ranging from 102.33 ± 0.58 cm (VAR18) to 156.67 ± 0.58 cm (VAR10) (Table 1). Tall genotypes, including VAR10, VAR13, and VAR2, contrasted markedly with the shorter statured VAR18, VAR17, and VAR16, reflecting substantial diversity in plant architecture that may influence lodging resistance, canopy structure, and biomass production. These differences are clearly reflected in Figure 1 by distinct median values among genotypes.

Significant diversity was also observed for panicle architecture and flag leaf characteristics. Panicle length varied from 21.77 ± 0.25 cm (VAR20) to 33.93 ± 1.95 cm (VAR12), suggesting marked differences in reproductive sink development. Flag leaf length ranged from 28.40 ± 0.95 cm (VAR18) to 53.03 ± 2.80 cm (VAR19), while flag leaf width ranged from 0.90 ± 0.00 cm to 1.80 ± 0.10 cm, resulting in corresponding variation in flag leaf area from 19.73 ± 0.32 cm^2^ (VAR18) to 49.70 ± 3.16 cm^2^ (VAR19). Similarly, the flag leaf length-to-width ratio varied considerably among genotypes, indicating substantial diversity in leaf architecture and canopy structure. Genotypes such as VAR19, VAR4, VAR21, and VAR2 consistently exhibited larger flag leaf areas, suggesting greater photosynthetic capacity and assimilate production during grain filling. These differences are further supported by the boxplots, which show clear separation among genotypes with relatively small within-genotype variation.

Tillering ability also differed significantly among the evaluated genotypes. The number of total tillers (NTT) ranged from 7.63 ± 0.95 (VAR20) to 15.57 ± 0.61 (VAR19), whereas productive tillers (NPT) varied from 7.80 ± 0.26 (VAR20) to 15.70 ± 0.44 (VAR16). Genotypes VAR16, VAR17, VAR19, and VAR15 consistently produced greater numbers of productive tillers, indicating superior sink establishment and greater yield potential. Figure 1 clearly illustrates these differences through distinct distributions and median values among the evaluated genotypes.

Substantial variation was likewise observed for sink size and biomass accumulation. The number of grains per panicle (NGPP), one of the principal yield-determining traits, ranged from 104.00 ± 1.73 grains (VAR18) to 267.33 ± 23.03 grains (VAR17), with VAR17, VAR16, VAR19, and VAR15 consistently recording higher grain numbers than the remaining accessions. Overall, the combined mean performance comparisons (Table 1) and boxplot distributions (Figure 1) demonstrate extensive phenotypic diversity in vegetative growth, phenology, leaf architecture, tillering ability, and sink development among the evaluated rice genotypes.

#### 2.1.2. Variation in Yield and Grain-Quality Traits Among Rice Genotypes

The mean comparison based on DMRT at the 5% significance level revealed substantial variation among the twenty-one rice genotypes for yield-related and grain-quality traits (Table 2). The presence of multiple significance groups for each trait reflects broad phenotypic diversity in grain productivity, yield components, and end-use quality, demonstrating the breeding potential of the evaluated germplasm.

**Figure 2:**
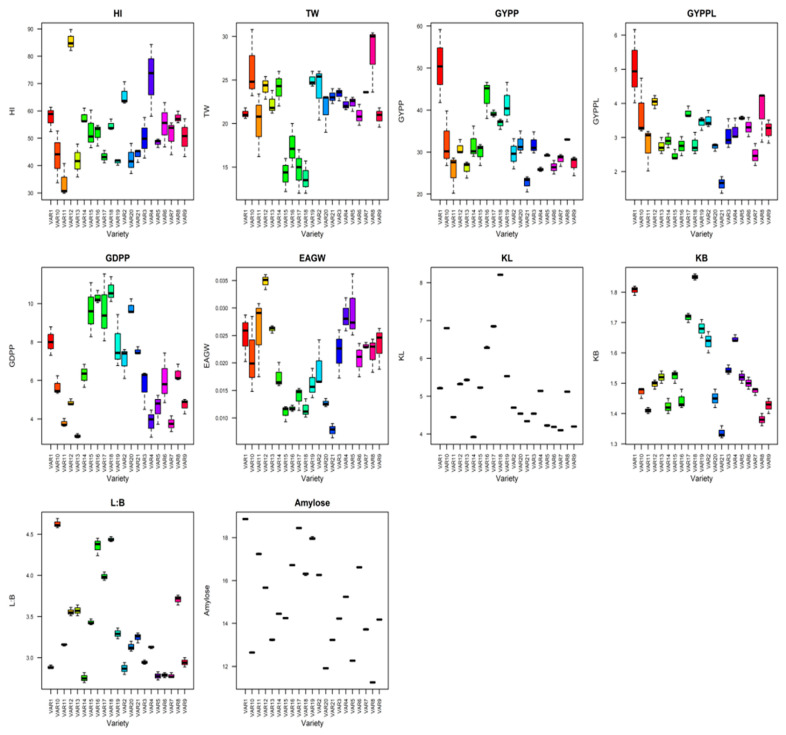
Distribution of key yield-related and quality traits among 21 genotypes. Boxplots represent variation in traits, including (HI, TW, GYPP, GYPPL, GDPP, EAGW, KL, KB, L:B, Amylose). Boxes represent the interquartile range (IQR), the horizontal line within each box indicates the median, whiskers extend to 1.5 × IQR and represent outliers.

Harvest index (HI), which reflects the efficiency of assimilate partitioning toward grain production, ranged from 32.33 ± 2.52% (VAR12) to 85.67 ± 2.52% (VAR11) (Table 2). Genotypes exhibiting higher harvest index values indicate greater efficiency in converting total biomass into economic yield. Test weight (TW), an important indicator of grain size and density, varied from 13.67 ± 1.15 g to 29.33 ± 1.15 g, with VAR8 producing the heaviest grains. The boxplots (Figure 2) clearly illustrate the distinct separation among genotypes for both harvest index and test weight while showing relatively narrow within-genotype variation, indicating consistent trait expression across replications.

Grain yield per hill (GYPP), the principal economic trait, exhibited considerable variation, ranging from 22.63 ± 1.55 g to 50.67 ± 4.93 g. Superior grain yield was recorded in VAR1, VAR16, VAR17, and VAR19, whereas comparatively lower yields were observed in VAR8, VAR11, and VAR20. Similarly, grain yield per productive panicle (GYPPL) ranged from 1.67 ± 0.21 g to 5.00 ± 0.58 g, indicating marked differences in panicle productivity among the evaluated accessions. The corresponding boxplots further confirm these differences by displaying clear separation between high- and low-yielding genotypes with limited overlap in their distributions.

Substantial diversity was also evident for sink efficiency traits. Grain density per panicle (GDPP) varied from 3.07 ± 0.15 to 10.87 ± 0.76 grains cm^−1^, while estimated average grain weight (EAGW) ranged from 0.008 ± 0.001 g to 0.035 ± 0.001 g, demonstrating significant variation in grain filling efficiency and individual grain weight among genotypes. Figure 2 highlights the pronounced variability for these traits, particularly grain density per panicle, which exhibited one of the widest phenotypic distributions across the evaluated germplasm.

Grain physical characteristics likewise exhibited broad phenotypic variation. Kernel length (KL) ranged from 3.93 ± 0.06 mm to 8.23 ± 0.06 mm, whereas kernel breadth (KB) varied between 1.34 ± 0.03 mm and 1.85 ± 0.01 mm, reflecting the presence of both slender- and bold-grained rice types. Consequently, the kernel length-to-breadth ratio (L) ranged from 2.77 ± 0.04 to 4.62 ± 0.05, demonstrating pronounced diversity in grain shape. Genotypes such as VAR18, VAR10, and VAR17 produced comparatively longer and more slender grains, characteristics generally preferred in premium rice markets. These differences are clearly illustrated in Figure 2, where distinct distributions and median values indicate stable expression of kernel morphology among genotypes.

Amylose content, an important determinant of cooking and eating quality, ranged from 11.27 ± 0.12% to 18.87 ± 0.15%. Higher amylose levels were recorded in VAR1, VAR17, VAR18, and VAR19, whereas VAR20 and VAR21 exhibited comparatively lower values, indicating considerable diversity in grain-quality attributes and potential consumer preferences. The boxplots further demonstrate the clear phenotypic differentiation among genotypes for amylose content, with relatively narrow interquartile ranges reflecting high experimental precision. Overall, the combined mean comparisons (Table 2) and the boxplot distributions (Figure 2) demonstrate extensive phenotypic variation for grain yield, yield components, grain morphology, and cooking quality traits among the evaluated rice genotypes. The consistently superior performance of VAR1, VAR16, VAR17, and VAR19 for several yield-related traits, together with the desirable grain-quality characteristics exhibited by VAR18, VAR17, and VAR1, highlights these accessions as valuable parental resources for breeding rice cultivars that combine high productivity with improved grain quality.

### 2.2. Analysis of Variance and Genetic Variability Parameters of Rice Genotypes

The analysis of variance (ANOVA) revealed highly significant (*p* < 0.001) genotypic differences for all twenty-one agronomic, yield, and grain-quality traits evaluated (Table 3), confirming the existence of substantial genetic variability among the rice accessions. The genotypes were particularly high in the mean square values of traits like number of grains per panicle (NGPP), biomass (BM), harvest index (HI), and grain yield per hill (GYPP), which indicated the strong genetic influence and the possibility of an effective selection.

The magnitude of genotypic coefficient of variation (GCV) and phenotypic coefficient of variation (PCV) varied among traits. High GCV and PCV values were recorded for grain density per panicle (GDPP) (35.06% and 37.25%), flag leaf area (FLA) (27.38% and 30.78%), number of grains per panicle (NGPP) (28.61% and 31.14%), biomass (BM) (26.37% and 28.12%), harvest index (HI) (21.22% and 23.98%), grain yield per hill (GYPP) (20.22% and 22.90%), productive tillers (NPT) (20.42% and 22.43%), and total tillers (NTT) (20.99% and 23.03%). These results indicate the presence of broad genetic variability and suggest substantial opportunities for effective phenotypic selection. The consistently small differences between GCV and PCV across traits further indicate that environmental effects on trait expression were relatively limited.

Broad-sense heritability estimates ranged from 62% for days to 50% flowering (DF50) to 100% for kernel length (KL) and amylose content, with most traits exhibiting high heritability (>80%). Very high heritability was observed for KL (100%), amylose content (100%), kernel length-to-breadth ratio (99%), kernel breadth (98%), grain density per panicle (89%), biomass (88%), number of grains per panicle (84%), plant height (83%), total tillers (83%), productive tillers (83%), flag leaf length (82%), and test weight (81%). These high estimates indicate that genetic factors predominantly controlled the expression of these traits and that phenotypic selection would be highly reliable.

Genetic advance (GA) and genetic advance as percentage of mean (GAM) provided additional insight into the expected response to selection. The highest GAM values were recorded for grain density per panicle (67.97%), number of grains per panicle (54.14%), biomass (50.96%), flag leaf area (50.19%), kernel length (43.65%), total tillers (39.40%), harvest index (38.69%), productive tillers (38.28%), and grain yield per hill (36.79%). The combination of high heritability with high GAM for these traits suggests that additive gene effects play a major role in their inheritance, making them highly amenable to direct phenotypic selection. Conversely, traits such as days to 50% flowering (12.61%), days to maturity (11.49%), and kernel breadth (18.25%) exhibited comparatively lower GAM despite moderate to high heritability, indicating relatively lower expected genetic gain from selection.

Overall, the combined evidence from ANOVA and genetic variability parameters demonstrates the presence of abundant exploitable genetic variation among the evaluated rice genotypes. In particular, number of grains per panicle, grain density per panicle, biomass, flag leaf area, grain yield per hill, tillering ability, and harvest index exhibited high genetic variability, high heritability, and high expected genetic advance, identifying these traits as the most promising selection criteria for developing high-yielding rice cultivars with improved agronomic performance.

### 2.3. Trait Association, Network Structure, and Path Analysis of Yield Architecture in Rice

#### 2.3.1. Trait Association and Correlation Analysis

Pearson’s correlation analysis at both the phenotypic (Table 4) and genotypic (Table 5) levels revealed complex relationships among vegetative, yield, and grain-quality traits, providing insights into the genetic architecture underlying grain yield in rice. In general, genotypic correlation coefficients were greater in magnitude than their corresponding phenotypic correlations, indicating that the intrinsic genetic associations among traits were stronger than those expressed phenotypically and that environmental variation slightly reduced the observable relationships.

Grain yield per hill (GYPP) exhibited significant positive associations with several economically important traits. At the phenotypic level, grain yield showed the strongest positive correlation with biomass (BM; *r* = 0.678), followed by grain density per panicle (GDPP; *r* = 0.521), kernel breadth (KB; *r* = 0.537), number of grains per panicle (NGPP; *r* = 0.499), amylose content (*r* = 0.496), kernel length (KL; *r* = 0.452), and grain yield per productive panicle (GYPPL; *r* = 0.605). Similar but generally stronger relationships were observed at the genotypic level, where biomass (*r* = 0.734), grain density per panicle (*r* = 0.635), number of grains per panicle (*r* = 0.622), kernel breadth (*r* = 0.607), amylose content (*r* = 0.564), grain yield per productive panicle (*r* = 0.560), kernel length (*r* = 0.514), and days to 50% flowering (*r* = 0.474) were positively associated with grain yield. These consistent relationships indicate that biomass accumulation, sink capacity, and grain characteristics collectively contribute to enhanced grain productivity.

Conversely, several morphological traits exhibited negative relationships with grain yield. Plant height, panicle length, flag leaf length, flag leaf area, flag leaf length-to-width ratio, and test weight were negatively correlated with grain yield at both phenotypic and genotypic levels. The strongest negative genotypic associations with grain yield were observed for flag leaf length (*r* = −0.496), panicle length (*r* = −0.483), flag leaf length-to-width ratio (*r* = −0.475), and plant height (*r* = −0.340), suggesting that increased vegetative growth alone did not necessarily translate into greater productivity under the present experimental conditions. Harvest index exhibited only a weak relationship with grain yield (phenotypic *r* = 0.077; genotypic *r* = −0.033), indicating that biomass accumulation rather than partitioning efficiency contributed more substantially to yield variation among the evaluated genotypes.

**Figure 3:**
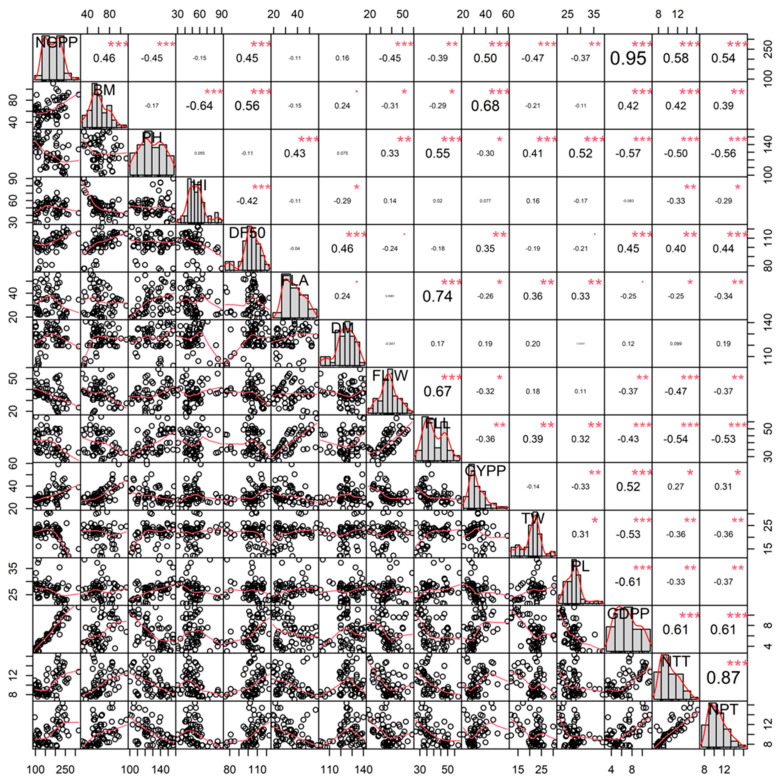
Pairwise correlation matrix showing relationships among the 15 major agronomic, yield, and grain-quality traits evaluated in 21 rice genotypes. Diagonal panels display the distribution (kernel density plots) of individual traits, with the lower triangle showing scatter plots with fitted regression lines and confidence intervals, which are used to display the relationships between trait pairs. The upper triangle displays Pearson correlation coefficients (*r*) along with their significance levels (* *p* < 0.05, ** *p* < 0.01, *** *p* < 0.001).

The correlation matrices also revealed several important interrelationships among yield-component traits. Biomass was strongly and positively associated with days to 50% flowering, number of grains per panicle, grain density per panicle, kernel dimensions, and amylose content (Figure 3), whereas grain density per panicle showed an exceptionally strong positive association with number of grains per panicle at both phenotypic (*r* = 0.948) and genotypic (*r* = 0.955) levels, confirming that grain number largely determines sink density. Likewise, total tillers and productive tillers exhibited a very strong positive association (*r* = 0.869 phenotypic; *r* = 0.964 genotypic), reflecting the close developmental relationship between tiller production and productive panicle formation.

**Figure 4:**
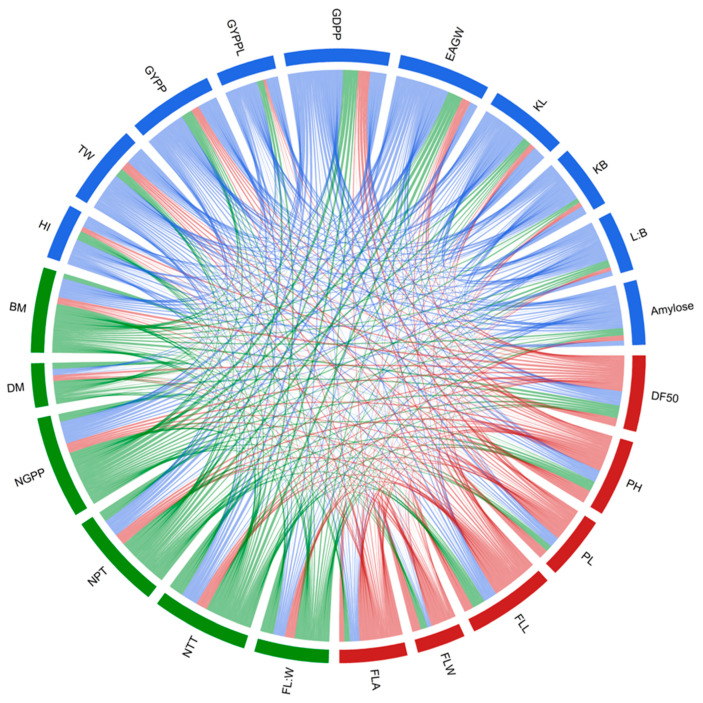
Integrated phenotypic connectivity network among agronomic, yield, and grain traits in rice germplasm. Phenotypic connectivity network among agronomic, yield, and grain traits in rice germplasm. The circular layout represents individual traits, while connecting ribbons (chords) indicate the strength and direction of associations between trait pairs derived from correlation analysis. The width of each ribbon reflects the magnitude of the relationship, with thicker connections indicating stronger associations.

The chord network (Figure 4) complements the correlation matrices by visually illustrating the strength and direction of trait interactions. Grain yield occupied a central position within the network and was connected predominantly with biomass, number of grains per panicle, grain density per panicle, kernel breadth, kernel length, grain yield per productive panicle, and amylose content through strong positive links. These interconnected traits formed a tightly associated yield module, indicating their coordinated contribution to grain production. In contrast, plant height, panicle length, and several flag leaf traits were connected to grain yield through comparatively weaker or negative associations, suggesting that these vegetative attributes contributed less directly to productivity. The close clustering of biomass and sink-related traits further emphasizes that yield improvement in the evaluated germplasm is primarily governed by enhanced assimilate production coupled with increased sink strength rather than by increased plant stature alone.

Overall, the combined phenotypic and genotypic correlation analyses, together with the correlation network, demonstrate that biomass accumulation, grain number, grain density, and kernel characteristics are the principal determinants of grain yield in the evaluated rice germplasm. These traits therefore represent reliable indirect selection criteria for developing high-yielding rice cultivars while simultaneously maintaining desirable grain quality.

#### 2.3.2. Path Coefficient and Multiple Regression Analyses

To distinguish direct effects from indirect associations observed in the correlation analysis, path coefficient analysis was performed by partitioning the correlation coefficients into their direct and indirect components (Table 6). The analysis identified biomass (BM) as the most influential determinant of grain yield, exhibiting the largest positive direct effect (0.693), confirming that dry matter accumulation is the principal driver of productivity in the evaluated rice germplasm. Number of grains per panicle (NGPP) also exerted a substantial positive direct effect (0.250) while contributing additional indirect effects through biomass and tillering traits, indicating that increased sink size enhances grain yield both directly and through improved assimilate accumulation.

Among the remaining traits, kernel breadth (0.336), harvest index (0.405), test weight (0.234), number of productive tillers (0.225), and total tillers (0.201) exhibited positive direct effects on grain yield. Nevertheless, their overall correlations with grain yield differed because of contrasting indirect effects mediated through other yield-related traits. Harvest index provides a clear example of this interaction, showing a relatively large positive direct effect but only a negligible total correlation with grain yield owing to its strong negative indirect effect via biomass. Likewise, kernel breadth contributed positively to grain yield both directly and indirectly through biomass and kernel-related traits, highlighting its dual contribution to grain productivity and grain quality.

Conversely, plant height exhibited the largest negative direct effect (−0.242), indicating that increased plant stature alone did not contribute positively to grain yield after accounting for the influence of other yield components. Panicle length displayed only a negligible negative direct effect (−0.004), whereas kernel length showed a small negative direct effect (−0.077), despite exhibiting a positive overall correlation with grain yield. This discrepancy indicates that the apparent contribution of kernel length to grain yield is primarily mediated through indirect effects involving biomass accumulation and kernel breadth rather than through an independent influence.

To further identify the most influential predictors of yield and grain quality, multiple regression analysis was performed (Table 7). For grain production, days to 50% flowering (β = 0.299; *p* = 0.008), plant height (β = −0.275; *p* = 0.015), and total tillers (β = 0.440; *p* = 0.018) were identified as significant predictors of grain number per panicle, whereas productive tillers and days to maturity were not statistically significant. These findings indicate that grain production is primarily governed by flowering duration, plant architecture, and tillering capacity, while maturity duration contributes comparatively less under the present experimental conditions.

For grain quality, kernel breadth emerged as the strongest positive predictor of amylose content (β = 1.751; *p* = 0.001), followed by the kernel length-to-breadth ratio (β = 2.111; *p* = 0.033). In contrast, kernel length (β = −2.720; *p* = 0.023) and test weight (β = −0.316; *p* = 0.003) exhibited significant negative effects on amylose content. These results indicate that grain physical characteristics are closely associated with cooking quality and can therefore be simultaneously exploited for quality improvement in rice breeding programs. Collectively, the path coefficient and multiple regression analyses consistently identified biomass accumulation, sink size, tillering ability, and kernel morphology as the principal determinants of grain productivity and grain quality. While biomass and grain number directly enhanced grain yield, kernel breadth and grain shape contributed not only to productivity but also to grain-quality attributes.

### 2.4. Multivariate Analysis of Phenotypic Diversity and Genotype Clustering

#### 2.4.1. Principal Component Analysis

**Figure 5:**
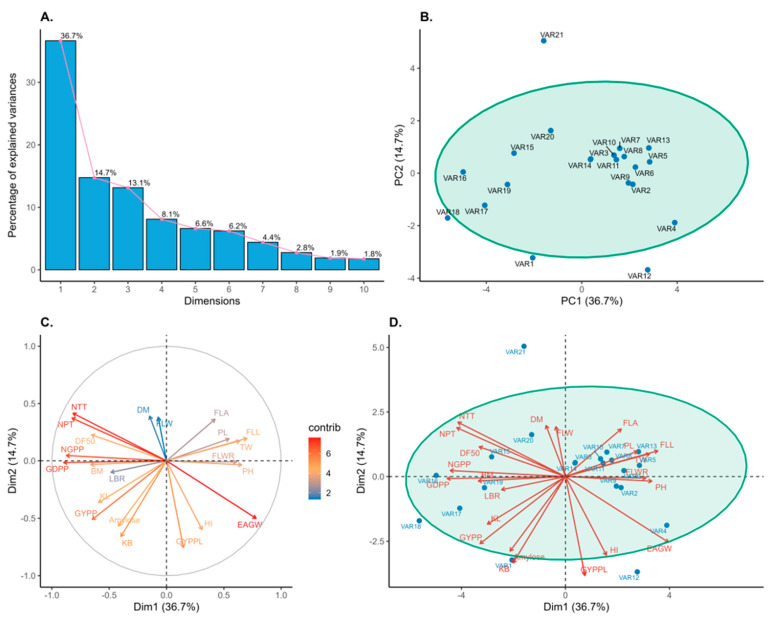
Principal component analysis (PCA) of 21 rice genotypes based on integrated agro-morphological, yield, and grain-quality traits. (**A**) Screen plot showing the percentage of total phenotypic variance explained by each principal component, with PC1 and PC2 accounting for 36.7% and 14.7% of the total variation, respectively (51.4% cumulative). (**B**) PCA score plot illustrating the distribution of the 21 rice genotypes on the PC1–PC2 plane, with the confidence ellipse representing the overall phenotypic dispersion. (**C**) Variable correlation circle showing the contribution and relationships of individual traits to the first two principal components; vector length indicates the strength of trait contribution, and the angle between vectors reflects trait correlations. (**D**) PCA biplot integrating genotype distribution and trait vectors, illustrating genotype–trait associations and the major sources of phenotypic variation among the evaluated rice genotypes.

Principal component analysis (PCA) was performed to characterize the multidimensional phenotypic diversity among the twenty-one rice genotypes using twenty-two agronomic and grain-quality traits (Figure 5). The screen plot (Figure 5A) indicated that the first two principal components accounted for 51.4% of the total phenotypic variation, with PC1 explaining 36.7% and PC2 explaining 14.7%, while the first three principal components together explained 64.6% of the total variation.

The distribution of genotypes on the PC1 vs. PC2 plane (Figure 5B) demonstrated substantial phenotypic diversity among the evaluated varieties. Most genotypes were dispersed throughout the confidence ellipse rather than forming compact clusters, indicating considerable genetic variability across the evaluated agronomic traits. Genotypes such as VAR4, VAR5, VAR7, VAR10, VAR13, and VAR21 occupied the positive side of PC1, whereas VAR16, VAR17, VAR18, and VAR19 were positioned on the negative side, reflecting contrasting phenotypic profiles. Notably, VAR21 was clearly separated from the remaining genotypes because of its exceptionally high PC2 score, suggesting a unique combination of morphological and yield-related characteristics. Similarly, VAR1 and VAR12, positioned toward the lower region of PC2, exhibited distinct phenotypic compositions compared with the remaining germplasm.

The variable correlation circle (Figure 5C) provided insights into the contribution and interrelationships among traits. Variables with long vectors and orientations close to the circumference contributed most strongly to the observed phenotypic variation. Yield-related traits, including estimated average grain weight (EAGW), grain yield per hill (GYPP), grain yield per plot (GYPPL), harvest index (HI), kernel breadth (KB), and amylose content, exhibited substantial contributions to PC1 and PC2, indicating that grain productivity and quality attributes collectively explained a considerable proportion of the overall diversity. Likewise, number of productive tillers (NPT), number of total tillers (NTT), number of grains per panicle (NGPP), days to 50% flowering (DF50), and grain density per panicle (GDPP) were strongly associated with the negative direction of PC1, suggesting that reproductive development and sink capacity represented another major source of phenotypic variation.

The relative orientation of trait vectors further revealed important biological relationships. Acute angles among vectors indicated positive correlations, whereas obtuse angles suggested negative associations. NPT, NTT, NGPP, DF50, and GDPP exhibited closely aligned vectors, indicating strong positive interrelationships among tillering capacity, flowering behavior, and grain production. Similarly, GYPP, GYPPL, HI, and EAGW clustered within the same quadrant, demonstrating that improvements in grain yield were closely associated with enhanced biomass partitioning efficiency and grain weight. Traits located near the origin, including leaf width (FLW) and days to maturity (DM), contributed relatively less to genotype discrimination because of their comparatively lower variability across the evaluated germplasm.

The integrated PCA biplot (Figure 5D) simultaneously illustrated genotype distribution and trait contributions, allowing direct identification of genotype–trait associations. Genotypes positioned in the direction of particular trait vectors exhibited relatively higher expression of those characteristics. Similarly, VAR4 was closely associated with effective average grain weight, harvest index, and grain yield-related traits, suggesting superior yield potential. VAR10, VAR13, and VAR7 were positioned near vectors representing vegetative growth traits, including flag leaf area, flag leaf length, and plant height, indicating greater vegetative vigor. Conversely, genotypes located on the negative side of PC1, including VAR16, VAR17, VAR18, and VAR19, were more closely associated with reproductive traits such as number of tillers, number of productive tillers, and grain density per panicle. The contrasting distribution of genotypes across the ordination space demonstrates that phenotypic variation was governed by multiple independent trait complexes rather than by a single dominant characteristic.

#### 2.4.2. Genotype Clustering Analysis

**Figure 6:**
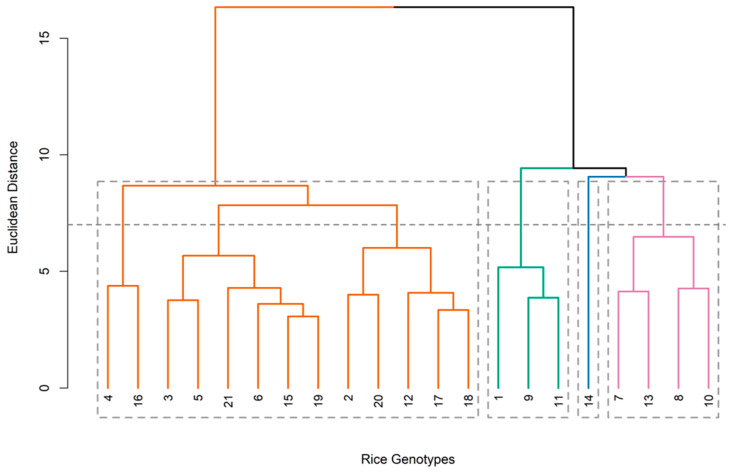
Hierarchical clustering dendrogram of 21 rice genotypes based on standardized agro-morphological, yield, and grain-quality traits using Euclidean distance and Ward’s linkage.

Hierarchical cluster analysis based on standardized agronomic, yield, and grain-quality traits grouped the 21 rice genotypes into four distinct clusters using Euclidean distance and Ward’s method (Figure 6; Table 8), demonstrating substantial phenotypic diversity within the population. Genotypes within the same cluster exhibited greater similarity across multiple traits than those in different clusters, confirming the effectiveness of multivariate clustering for identifying phenotypically related materials.

Cluster I comprised the largest proportion of genotypes, indicating relatively high similarity in overall agronomic and yield performance and suggesting a comparatively narrow phenotypic range among these varieties. In contrast, Clusters II and IV contained fewer genotypes, while Cluster III consisted of a single genotype (VAR14), highlighting its distinct phenotypic profile. Such isolated genotypes are valuable in breeding because they may possess unique trait combinations or favorable alleles that can broaden the genetic base of future breeding populations.

The major separation of clusters at higher Euclidean distances indicates substantial divergence in the combined expression of flowering time, biomass accumulation, yield components, and grain-quality traits. This clustering pattern closely agreed with the PCA results, where genotypes with similar multivariate trait profiles were grouped together, reinforcing the reliability of the observed population structure. From a breeding perspective, the identified clusters provide a practical framework for parental selection. Crosses between genotypes from genetically distant clusters are expected to maximize genetic recombination and increase the probability of obtaining superior transgressive segregants with improved yield and grain quality.

### 2.5. Genotype Ranking and Selection Index Analysis

The multi-trait selection index (Table 9), integrating grain yield per plant (GYPP), grain amylose content, and length-to-breadth (L) ratio, was employed to rank the 21 rice genotypes based on their overall agronomic and grain-quality performance. This integrated approach enabled simultaneous evaluation of productivity and market-preferred grain characteristics, thereby facilitating balanced genotype selection rather than relying on yield alone.

Based on the selection index, VAR16 ranked first, exhibiting a desirable combination of high grain yield (43.27 g plant^−1^) and superior grain architecture (L ratio = 4.36), indicating an excellent balance between productivity and grain quality. Although VAR1 recorded the highest grain yield (50.47 g plant^−1^), it ranked third overall because of its comparatively lower grain slenderness, illustrating the importance of considering multiple traits during genotype selection. Likewise, VAR12, VAR17, and VAR18 consistently combined favorable yield performance with desirable grain characteristics, making them promising candidates for future breeding programs. In contrast, VAR21 and VAR5 ranked lowest owing to their relatively poor performance across the evaluated traits. The selection index results were generally consistent with the PCA and hierarchical clustering analyses, where the top-ranked genotypes were positioned within phenotypically distinct groups characterized by favorable combinations of yield and grain-quality attributes.

## 3. Discussion

The present study demonstrated substantial phenotypic variation among the 21 rice genotypes for agronomic, yield, and grain-quality traits, indicating that the evaluated germplasm possesses sufficient diversity for effective phenotypic selection. The highly significant genotypic differences observed across all measured traits, together with moderate-to-high GCV, PCV, heritability, and genetic advance estimates for major yield components, suggest that much of the observed variation was genetically controlled rather than environmentally influenced. Particularly, biomass, number of grains per panicle, grain density per panicle, productive tillers, and grain yield exhibited high heritability coupled with high genetic advance, indicating the predominance of additive gene action and supporting their use as reliable selection criteria in early-generation breeding [17].

These findings show a broad genetic base within the studied germplasm, offering considerable scope for selection and improvement of both yield and quality traits. Similar findings were reported by Pandey et al. and Asante et al. [18,19], who found high PCV and GCV and high heritability and genetic advance in plant height, grain yield per hill, harvest index, and biological yield among 40 rice genotypes. Similarly, Asante et al. and Tuhina-Khatun et al. [19,20] documented considerable variation and high heritability for filled grains per panicle and yield per plant in upland rice germplasm. Salem et al. and Debsharma et al. [21,22] also verified the significant phenotypic diversity of rice genotypes, whereas the elements of yield contribute a significant part to overall variation. The existence of such appreciable variability in the current set of genotypes provides a strong foundation for genetic improvement and helps overcome the genetic bottlenecks and yield plateaus experienced in many rice-growing regions following the green revolution [23,24].

The integration of correlation, network analysis, path analysis, and multiple regression provided a more comprehensive understanding of yield architecture than any individual statistical approach alone. Correlation analysis identified biomass, grain number, kernel breadth, and amylose content as positively associated with grain yield, whereas path analysis distinguished true causal contributors from indirect associations. Biomass exhibited the largest positive direct effect on grain yield, confirming that greater assimilate production remains the primary physiological driver of productivity. Grain number per panicle also contributed directly while reinforcing yield indirectly through biomass accumulation, highlighting the complementary roles of source strength and sink capacity during grain filling. The correlation network further demonstrated that these traits form a tightly interconnected module governing yield formation, illustrating that rice productivity results from coordinated interactions among multiple physiological processes rather than from isolated traits.

An interesting outcome of this study was the weak overall association between harvest index and grain yield despite the relatively large positive direct effect identified through path analysis. This apparent inconsistency indicates that the positive influence of harvest index was offset by strong negative indirect effects, particularly through biomass, resulting in an overall near-zero correlation. Therefore, the present findings suggest that variation in grain yield among these genotypes was driven primarily by differences in total biomass production rather than assimilate partitioning efficiency.

Another noteworthy finding was the positive association between amylose content and grain yield. This contrasts with the commonly reported yield–quality trade-off in rice, where increasing grain quality is often accompanied by reduced productivity. The present germplasm may therefore contain genotypes capable of partially overcoming this trade-off. A possible physiological explanation is that superior photosynthetic capacity and efficient carbohydrate production simultaneously supported grain filling and starch biosynthesis, enabling both higher yield and moderate-to-high amylose accumulation. Although the underlying molecular mechanisms were beyond the scope of this study, the observed relationship suggests that some of the evaluated genotypes may partially overcome the conventional yield–quality trade-off. Validation through genomic and multi-environment studies will be necessary to confirm this hypothesis. The present findings strongly corroborate Bagheri et al. and Idris et al. [25,26], who reported high positive correlations of grain yield with panicle length (*r* = 0.818), filled grains per panicle (*r* = 0.790), and panicles per plant, with path analysis identifying panicle length as having the maximum direct effect (0.510). Oladosu et al. [27] similarly highlighted the prominent direct effects of filled grains per panicle and grain weight per hill under tropical conditions. By integrating correlation with path analysis within a network perspective, the current study overcomes the limitations of conventional bivariate methods that cannot distinguish direct from indirect effects, thereby offering more reliable selection criteria for breeders [28,29].

The multivariate analyses further demonstrated that rice phenotypic diversity was governed by multiple independent trait complexes. Although the first two principal components explained 51.4% of the total variation, this proportion is typical for complex quantitative traits controlled by numerous genes [30,31]. The remaining variation distributed across subsequent components reflects the polygenic nature of rice yield and grain-quality traits rather than the weakness of the PCA model. Importantly, PCA consistently identified biomass accumulation, grain number, tillering ability, harvest index, and grain-quality traits as the principal sources of phenotypic differentiation. Hierarchical clustering produced four distinct phenotypic groups, largely consistent with PCA, indicating that the evaluated genotypes differ substantially in their overall trait combinations. The agreement between these independent multivariate approaches increases confidence in the observed population structure. These multivariate results are in line with the studies of Maji and Shaibu [32], who found that the first two PCs explained 78% of the variation in Nigerian rice germplasm, while cluster analysis effectively separated different eco-types. Salem et al. [21] reported that PC1 and PC2 accounted for 66.1% of variation, driven primarily by yield and its components. Debsharma et al. [22] also utilized PCA and clustering alongside MGIDI for effective genotype discrimination. The integrated multivariate framework adopted in this study provides a more comprehensive understanding of trait interrelationships and genotypic structure compared to isolated analytical tools, facilitating the identification of complementary parents capable of producing transgressive segregants with enhanced yield potential [33,34].

The multi-trait selection index further identified VAR16, VAR1, VAR17, VAR18, and VAR12 as promising breeding materials because they combined favorable productivity with desirable grain characteristics. However, these rankings should be interpreted within the context of the present experimental conditions. Since the evaluation was conducted at a single location during one growing season, genotype × environment interactions could not be quantified. Therefore, the observed rankings represent performance under the tested environment rather than stable superiority across diverse production conditions. Multi-location and multi-season evaluations are necessary before these genotypes can be recommended for cultivar release or routine use as elite breeding parents [35,36,37]. Unlike studies that apply multivariate methods independently, the present work integrated quantitative genetic parameters, correlation networks, path analysis, PCA, hierarchical clustering, and a multi-trait selection index within a unified analytical framework. This integration enabled the identification of key yield-driving traits, elucidated their physiological relationships, classified phenotypically diverse genotypes, and ranked breeding materials simultaneously [38].

Several limitations should be considered when interpreting the present findings. First, only 21 rice genotypes were evaluated under a single environment, limiting inference regarding genotype stability and genotype × environment interactions. Second, the PCA explained approximately half of the total phenotypic variation within the first two components, indicating that additional quantitative traits also contributed to overall diversity. Furthermore, the PCA and cluster analysis were not validated using bootstrap resampling or an independent dataset because only 21 genotypes were evaluated. Therefore, the observed clustering patterns should be considered preliminary and require confirmation in larger germplasm collections. Third, the clustering analysis represents phenotypic similarity rather than direct genetic relationships because molecular marker information was not included. Finally, although the integrated analytical framework effectively identified promising breeding materials and key yield determinants, the conclusions are limited to the evaluated germplasm and should not be generalized to the broader rice gene pool.

Future research should therefore expand the germplasm panel, conduct multi-location and multi-season trials, incorporate genomic and marker-assisted analyses, and validate the proposed parental combinations through crossing experiments. Combining phenotypic selection with genomic information will improve prediction accuracy for complex traits and accelerate the development of high-yielding rice cultivars with superior grain quality adapted to subtropical production environments.

## 4. Materials and Methods

### 4.1. Experimental Location and Site Description

The study was conducted during a single wet-season growing cycle (Kharif season) at a single experimental location, namely the research farm of the Department of Genetics and Plant Breeding, Sam Higginbottom University of Agriculture, Technology and Sciences (SHUATS), Prayagraj, Uttar Pradesh, India. Thus, all phenotypic evaluations were performed under one environment (one location × one year).

The experimental site is located at 25.57° N latitude and 81.51° E longitude, at an altitude of 98 m above mean sea level, approximately 6 km from the right bank of the Yamuna River. The area belongs to the North Alluvial Plain Zone and forms part of the Upper Gangetic Plain agro-climatic region.

The climate of Prayagraj is subtropical, characterized by distinct seasonal variation. Winter temperatures (December–January) typically range from 3 to 5 °C, whereas summer temperatures (May–June) may reach 45–48 °C. The region receives an average annual rainfall of approximately 850–1100 mm, with nearly all precipitation occurring during the southwest monsoon season, providing favorable conditions for lowland rice cultivation.

### 4.2. Experimental Design and Crop Management

The experiment was evaluated using a randomized complete block design (RCBD) with three replications. The 21 rice genotypes were selected to represent a diverse panel of lowland rice germplasm, including advanced breeding lines and commercially released varieties, with broad variation in agronomic performance, yield potential, and grain-quality traits. The ecological classification and material type of each accession are presented in Supplementary Table S1.

The experimental field comprised a gross area of 520 m^2^ with a net cultivated area of 480 m^2^. Each experimental plot measured 1.0 × 8.0 m. Twenty-one-day-old healthy seedlings were transplanted manually at a spacing of 20 cm between rows and 15 cm between plants to maintain a uniform plant population across all plots.

A recommended fertilizer dose of @120:60:60 kg ha^−1^ NPK was uniformly applied to all experimental plots. Nitrogen was supplied in split applications following standard rice production practices, whereas the full doses of phosphorus and potassium were incorporated as basal fertilizers before transplanting. Irrigation was provided uniformly throughout the crop growth period to maintain an adequate standing water level suitable for lowland rice cultivation, thereby minimizing moisture stress.

Standard agronomic management practices were adopted uniformly for all treatments. Weeds were controlled through timely manual weeding as required. Insect pests were monitored regularly throughout the growing season and managed according to the principles of integrated pest management (IPM) [39]. When pest populations exceeded the economic threshold level, insecticidal applications of cypermethrin and lambda-cyhalothrin were applied at the recommended field dosages to effectively control major insect pests. Rice blast (*Magnaporthe oryzae*) and sheath blight (*Rhizoctonia solani*) were managed through timely applications of recommended fungicides, including tricyclazole for blast and validamycin for sheath blight, following standard crop protection recommendations [39].

### 4.3. Data Collection and Quality Analysis

Five representative plants were randomly selected from the central rows of each experimental plot (replication) for every genotype to record agronomic, yield-related and grain-quality traits, thereby minimizing border effects. The recorded traits included days to 50% flowering, plant height (cm), flag leaf length (cm), flag leaf width (cm), number of total tillers, number of productive tillers, panicle length (cm), flag leaf area (cm^2^) number of grains per panicle, days to maturity, biomass (g), harvest index (%), test weight (g), and grain yield per hill (g).

#### 4.3.1. Assessment of Flag Leaf Area

Flag leaf area (FLA, cm^2^) was estimated using the empirical equation proposed for rice:

FLA=0.75×FLL×FLW

where 0.75 is the correction factor (K) commonly used for rice leaves [40].

The flag leaf length-to-width ratio (FL:W) was calculated as:

$$FL:W=\frac{FLL}{FLW}$$

#### 4.3.2. Assessment of Yield-Related Parameters

Grain yield per productive panicle (GYPPL, g) was calculated as:

$$GYPPL=\frac{GYPP}{NPT}$$

Gain density per panicle (GDPP) was estimated as:

$$GDPP=\frac{NGPP}{PL}$$

The average estimated grain weight (EAGW, g grain^−1^) was calculated as:

$$EAGW=\frac{GYPP}{NGPP\times NPT}$$

#### 4.3.3. Assessment of Harvest Index

Harvest index (HI, %) was calculated by using the following formula:

$$HI\left(\%\right)=\frac{Grain\;yield\;(economic\;yield)}{Biological\;yield\;(grain\;yield+straw)}\times 100$$

where biological yield represents the sum of grain and straw yield.

### 4.4. Seed Trait Measurement

Kernel length (KL, mm) and kernel breadth (KB, mm) were measured on ten randomly selected fully developed grains from each genotype using a digital Vernier caliper with a precision of 0.01 mm. Measurements were performed on dehulled rice grains (with bran intact). The kernel length-to-breadth ratio (L:B) was calculated as in [41].

$$\mathrm{Seed}\;\mathrm{Length}\;\mathrm{Width}\;\mathrm{Ratio}=\frac{\left.\mathrm{Seed}\;\mathrm{Length}\;(\mathrm{mm}\right)}{\mathrm{Seed}\;\mathrm{breadth}\;(\mathrm{mm})}$$

The quality of grains was determined by the amylose content, which was estimated by the standard iodine colorimetric method by Rao et al. [42]. For each genotype, three independent technical replicates were analyzed from representative grain samples. Starch was extracted from finely ground rice flour, reacted with iodine reagent, and the absorbance of the starch–iodine complex was measured spectrophotometrically at the recommended wavelength. Amylose concentration was calculated from a standard calibration curve prepared using analytical-grade potato amylose standards and expressed as a percentage of total starch. The mean value of the three technical replicates was used for statistical analysis to ensure the repeatability and reliability of the measurements [42].

### 4.5. Statistical Analysis

Data analysis was conducted by using R software (R version 4.6.1). Analyses of variance and mean comparison using Duncan’s Multiple Range Test (DMRT) were analyzed by using the agricolae package. Pearson correlation analysis was carried out by using the *ggally* package, and the circle correlation network analysis was performed by using the *Circilize* package. PCA and its visualization were performed using the *factoextra* package.

## 5. Conclusions

This study revealed substantial phenotypic and genetic variability among 21 rice genotypes for agro-morphological, yield, and grain-quality traits, confirming their potential for rice improvement. High heritability coupled with high genetic advance for traits such as biomass, number of grains per panicle, grain density per panicle, tillering ability, and grain yield indicated that these traits are amenable to effective phenotypic selection. Correlation, path coefficient, and regression analyses consistently identified biomass accumulation, grain number per panicle, tillering ability, and kernel morphology as the major determinants of grain productivity and quality. Principal component analysis explained 51.4% of the total phenotypic variation in the first two components, while hierarchical clustering grouped the genotypes into four distinct clusters, providing a basis for selecting diverse parents. The multi-trait selection index identified VAR16 as the most promising genotype, followed by VAR1, VAR17, VAR18, and VAR12. Overall, these findings provide a robust framework for selecting superior parents and key target traits to accelerate the development of high-yielding, good-quality rice cultivars adapted to subtropical environments.

## Figures and Tables

**Table 1:** Mean performance (mean ± SD) of 21 rice genotypes for agro-morphological, phenological, and vegetative traits evaluated under field conditions. Means within each column followed by different lowercase letters differ significantly according to DMRT at *p* < 0.05.

Variety	DF50 (Days)	PH (cm)	PL (cm)	FLL (cm)	FLW (cm)	FLA (cm^2^)	FL:W (cm)	NTT	NPT	NGPP
VAR1	107.67 ^ab^ ± 4.50	123.30 ^f^ ± 3.44	25.10 ^defgh^ ± 0.74	34.97 ^def^ ± 3.25	1.30 ^bc^ ± 0.00	34.09 ^fg^ ± 3.18	26.9 0^efg^ ± 2.50	10.13 ^def^ ± 0.41	10.07 ^efgh^ ± 0.34	201.30 ^bcdef^ ± 9.92
VAR2	103.67 ^ab^ ± 3.30	149.80 ^ab^ ± 0.92	26.67 ^cdefg^ ± 0.71	52.57 ^a^ ± 3.53	1.10 ^cdef^ ± 0.14	43.75 ^abcde^ ± 8.78	48.14 ^ab^ ± 2.71	8.07 ^g^ ± 1.11	8.47 ^hij^ ± 1.09	188.17 ^cdef^ ± 22.39
VAR3	109.00 ^ab^ ± 10.03	124.17 ^f^ ± 0.82	24.83 ^efgh^ ± 0.16	40.47 ^cd^ ± 1.94	1.20 ^bcd^ ± 0.00	36.42 ^defg^ ± 1.74	33.72 ^cde^ ± 1.61	10.27 ^de^ ± 0.77	10.47 ^defg^ ± 0.50	142.03 ^gh^ ± 20.88
VAR4	84.00 ^d^ ± 11.34	139.43 ^bcd^ ± 1.10	30.13 ^bc^ ± 5.88	46.90 ^ab^ ± 1.57	1.13 ^cde^ ± 0.13	39.68 ^cdef^ ± 4.85	42.65 ^abc^ ± 8.49	8.07 ^g^ ± 0.41	8.13 ^ij^ ± 0.52	112.00 ^h^ ± 4.08
VAR5	104.00 ^ab^ ± 2.94	144.87 ^bc^ ± 1.12	26.97 ^cdefg^ ± 0.08	46.93 ^ab^ ± 0.26	1.27 ^bc^ ± 0.19	44.61 ^abcd^ ± 6.78	37.97 ^cd^ ± 6.18	8.40 ^fg^ ± 0.43	8.20 ^ij^ ± 0.16	123.63 ^h^ ± 17.10
VAR6	99.33 ^bc^ ± 4.78	131.03 ^def^ ± 14.86	27.00 ^cdefg^ ± 0.47	50.43 ^ab^ ± 1.68	1.37 ^b^ ± 0.03	51.64 ^a^ ± 0.17	36.99 ^cd^ ± 2.57	8.07 ^g^ ± 0.25	8.00 ^ij^ ± 0.16	163.27 ^fg^ ± 31.75
VAR7	109.00 ^ab^ ± 5.35	140.63 ^bcd^ ± 0.97	28.67 ^bcd^ ± 0.02	35.87 ^def^ ± 1.33	0.93 ^ef^ ± 0.04	25.13 ^hi^ ± 1.95	38.49 ^cd^ ± 1.66	9.73 ^efg^ ± 1.16	11.50 ^de^ ± 1.44	107.50 ^h^ ± 9.39
VAR8	104.33 ^ab^ ± 3.68	125.87 ^ef^ ± 2.50	27.07 ^cdefg^ ± 1.05	47.20 ^ab^ ± 4.09	1.20 ^bcd^ ± 0.06	42.23 ^bcdef^ ± 0.85	39.75 ^bc^ ± 6.12	8.17 ^g^ ± 0.60	9.07 ^ghij^ ± 1.79	171.57 ^efg^ ± 11.44
VAR9	88.67 ^cd^ ± 8.18	109.40 ^hi^ ± 1.56	29.50 ^bc^ ± 0.35	36.33 ^def^ ± 2.63	1.00 ^def^ ± 0.12	27.46 ^ghi^ ± 5.60	36.75 ^cd^ ± 3.53	8.20 ^g^ ± 0.33	8.47 ^hij^ ± 0.23	139.27 ^gh^ ± 10.71
VAR10	106.67 ^ab^ ± 1.25	156.70 ^a^ ± 0.72	32.00 ^ab^ ± 2.52	49.03 ^ab^ ± 1.00	1.27 ^bc^ ± 0.05	46.58 ^abc^ ± 1.83	38.77 ^cd^ ± 1.80	8.47 ^efg^ ± 0.52	8.67 ^hij^ ± 0.38	182.70 ^def^ ± 27.34
VAR11	103.67 ^ab^ ± 3.30	142.30 ^bcd^ ± 8.70	28.57 ^bcde^ ± 0.48	38.60 ^de^ ± 2.82	0.97 ^ef^ ± 0.05	27.92 ^ghi^ ± 1.59	40.13 ^bc^ ± 4.53	9.73 ^efg^ ± 0.52	9.33 ^fghij^ ± 0.47	108.00 ^h^ ± 5.31
VAR12	90.00 ^cd^ ± 9.27	142.23 ^bcd^ ± 11.00	23.93 ^fgh^ ± 0.20	33.63 ^efg^ ± 1.04	0.90 ^f^ ± 0.00	22.70 ^hi^ ± 0.71	37.37 ^cd^ ± 1.17	8.33 ^fg^ ± 0.47	7.67 ^j^ ± 0.19	116.10 ^h^ ± 3.34
VAR13	107.33 ^ab^ ± 5.44	147.50 ^abc^ ± 7.45	33.77 ^a^ ± 2.64	48.87 ^ab^ ± 7.65	0.97 ^ef^ ± 0.05	35.65 ^efg^ ± 6.96	50.35 ^a^ ± 6.07	9.40 ^efg^ ± 0.43	9.53 ^fghi^ ± 0.34	104.80 ^h^ ± 5.37
VAR14	91.00 ^cd^ ± 7.12	119.10 ^fgh^ ± 1.05	26.63 ^cdefg^ ± 0.76	33.13 ^efg^ ± 1.98	0.93 ^ef^ ± 0.05	23.13 ^hi^ ± 0.64	35.68 ^cde^ ± 3.67	11.60 ^cd^ ± 1.75	10.93 ^def^ ± 0.68	167.07 ^fg^ ± 11.43
VAR15	110.67 ^ab^ ± 6.02	110.97 ^ghi^ ± 0.48	23.60 ^gh^ ± 0.39	33.13 ^efg^ ± 1.98	0.97 ^ef^ ± 0.12	24.08 ^hi^ ± 3.90	34.82 ^cde^ ± 4.77	11.63 ^cd^ ± 0.87	12.07 ^cd^ ± 0.74	227.50 ^bc^ ± 23.47
VAR16	106.00 ^ab^ ± 6.38	107.30 ^hi^ ± 3.32	22.70 ^h^ ± 0.82	32.23 ^efg^ ± 1.35	0.97 ^ef^ ± 0.05	23.39 ^hi^ ± 1.74	33.40 ^cde^ ± 1.75	14.27 ^ab^ ± 0.62	15.73 ^a^ ± 0.47	234.10 ^b^ ± 10.43
VAR17	116.00 ^a^ ± 0.82	136.53 ^cde^ ± 2.15	28.40 ^bcde^ ± 0.79	33.73 ^efg^ ± 2.10	1.37 ^b^ ± 0.05	34.51 ^fg^ ± 0.98	24.77 ^fg^ ± 2.42	12.13 ^c^ ± 0.57	10.53 ^defg^ ± 0.52	273.30 ^a^ ± 33.28
VAR18	114.67 ^a^ ± 5.25	102.07 ^i^ ± 1.48	22.60 ^h^ ± 0.55	28.10 ^g^ ± 0.79	0.93 ^ef^ ± 0.04	19.64 ^i^ ± 0.43	30.22 ^def^ ± 2.28	12.53 ^c^ ± 1.32	13.27 ^bc^ ± 0.90	241.13 ^ab^ ± 6.08
VAR19	115.67 ^a^ ± 0.47	127.47 ^ef^ ± 7.72	27.50 ^cdef^ ± 0.28	30.83 ^fg^ ± 2.71	1.27 ^bc^ ± 0.09	29.39 ^gh^ ± 4.10	24.40 ^fg^ ± 2.11	12.23 ^c^ ± 0.76	12.07 ^cd^ ± 1.05	216.50 ^bcd^ ± 29.05
VAR20	111.00 ^ab^ ± 0.82	121.37 ^fg^ ± 1.14	21.97 ^h^ ± 0.38	45.33 ^bc^ ± 2.43	0.90 ^f^ ± 0.00	30.60 ^gh^ ± 1.63	50.37 ^a^ ± 2.68	12.67 ^bc^ ± 0.96	11.73 ^cde^ ± 0.84	214.83 ^bcd^ ± 5.61
VAR21	105.33 ^ab^ ± 1.77	123.00 ^f^ ± 4.24	27.97 ^cde^ ± 0.39	35.83 ^def^ ± 0.62	1.83 ^a^ ± 0.13	49.33 ^ab^ ± 4.25	19.61 ^g^ ± 0.97	15.60 ^a^ ± 0.57	14.00 ^b^ ± 0.82	210.60 ^bcde^ ± 3.10
CV	8.48%	12.02%	11.60%	18.49%	19.14%	22.48%	21.85%	22.20%	20.19%	26.65%

DF50 (days to 50% flowering), number of productive tillers (NPT), plant height (PH), panicle length (PL), flag leaf length (FLL), flag leaf width (FLW), flag leaf area (FLA), flag leaf length: width ratio (FL:W), number of total tillers (NTT), number of grains per panicle (NGPP), days to maturity (DM), bio mass (BM), harvest index (HI), grain yield per productive panicle (g panicle^−1^) (GYPPL), grain density per panicle (grains cm^−1^) (GDPP), estimated average grain weight (g grain^−1^)(EAGW), test weight (TW), grain yield per plant (GYPP), kernel length (KL), kernel breadth (KB), kernel length:kernel breadth (L:B).

**Table 2:** Mean performance (mean ± SD) of 21 rice genotypes for yield-related and quality traits evaluated under field conditions. Means within each column followed by different lowercase letters differ significantly according to DMRT at *p* < 0.05.

Variety	DM	BM (gm)	HI (%)	TW (gm)	GYPP	GYPPL	GDPP	EAGW	KL (mm)	KB (mm)	L:B	Amylose (%)
VAR1	134.67 ^abc^ ± 3.68	87.30 ^b^ ± 6.92	57.54 ^cd^ ± 3.78	21.13 ^de^ ± 0.50	50.47 ^a^ ± 7.10	5.04 ^a^ ± 0.88	8.04 ^b^ ± 0.61	0.0250 ^bcde^ ± 0.0035	5.22 ^h^ ± 0.02	1.81 ^b^ ± 0.01	2.89 ^i^ ± 0.02	18.87 ^a^ ± 0.03
VAR2	139.67 ^a^ ± 1.25	45.37 ^jk^ ± 6.58	65.73 ^bc^ ± 3.50	23.93 ^bcd^ ± 2.51	29.60 ^efg^ ± 2.94	3.51 ^bcde^ ± 0.20	7.04 ^bcd^ ± 0.67	0.0191 ^efgh^ ± 0.0036	4.70 ^j^ ± 0.01	1.64 ^e^ ± 0.03	2.87 ^ij^ ± 0.06	16.26 ^g^ ± 0.02
VAR3	128.33 ^bcdefg^ ± 3.86	64.23 ^efg^ ± 5.85	47.49 ^defg^ ± 6.07	23.40 ^bcde^ ± 0.59	31.87 ^defg^ ± 2.13	3.06 ^cdef^ ± 0.36	5.72 ^def^ ± 0.87	0.0220 ^defg^ ± 0.0036	4.54 ^k^ ± 0.02	1.54 ^f^ ± 0.01	2.94 ^i^ ± 0.02	14.24 ^kl^ ± 0.02
VAR4	104.00 ^j^ ± 1.63	36.83 ^k^ ± 6.32	72.06 ^b^ ± 10.81	22.20 ^cde^ ± 0.59	25.87 ^fgh^ ± 0.41	3.20 ^bcdef^ ± 0.26	3.83 ^gh^ ± 0.59	0.0286 ^bc^ ± 0.0025	5.14 ^i^ ± 0.01	1.65 ^de^ ± 0.01	3.12 ^h^ ± 0.01	15.24 ^i^ ± 0.01
VAR5	130.33 ^bcde^ ± 4.50	60.50 ^fgh^ ± 1.39	48.40 ^defg^ ± 1.29	22.33 ^cde^ ± 0.68	29.27 ^efg^ ± 0.25	3.57 ^bcde^ ± 0.04	4.58 ^fgh^ ± 0.63	0.0295 ^ab^ ± 0.0048	4.23 ^n^ ± 0.02	1.52 ^fg^ ± 0.02	2.78 ^jk^ ± 0.04	12.26 ^p^ ± 0.02
VAR6	120.33 ^fgh^ ± 1.25	48.47 ^ij^ ± 5.61	55.16 ^cde^ ± 6.64	20.93 ^de^ ± 0.98	26.40 ^efgh^ ± 1.31	3.30 ^bcdef^ ± 0.23	6.03 ^cdef^ ± 1.07	0.0207 ^defg^ ± 0.0025	4.18 ^o^ ± 0.01	1.50 ^gh^ ± 0.02	2.79 ^jk^ ± 0.03	16.62 ^f^ ± 0.02
VAR7	129.33 ^bcdef^ ± 8.22	55.63 ^fghij^ ± 3.63	51.14 ^defg^ ± 5.12	23.60 ^bcde^ ± 0.00	28.27 ^efgh^ ± 1.20	2.49 ^f^ ± 0.26	3.75 ^gh^ ± 0.33	0.0231 ^cdef^ ± 0.0004	4.10 ^p^ ± 0.02	1.47 ^hi^ ± 0.01	2.78 ^jk^ ± 0.03	13.73 ^m^ ± 0.03
VAR8	131.67 ^abcd^ ± 2.05	57.50 ^fghi^ ± 1.61	57.56 ^cd^ ± 1.78	28.00 ^a^ ± 3.12	33.07 ^de^ ± 0.09	3.77 ^bc^ ± 0.65	6.34 ^cde^ ± 0.36	0.0219 ^defg^ ± 0.0026	5.12 ^i^ ± 0.02	1.38 ^l^ ± 0.02	3.71 ^d^ ± 0.05	11.26 ^r^ ± 0.01
VAR9	114.33 ^hi^ ± 4.19	54.10 ^ghij^ ± 2.64	50.41 ^defg^ ± 5.67	20.80 ^de^ ± 0.91	27.13 ^efgh^ ± 1.95	3.21 ^bcdef^ ± 0.28	4.72 ^fg^ ± 0.33	0.0233 ^bcdef^ ± 0.0032	4.20 ^no^ ± 0.01	1.43 ^jk^ ± 0.02	2.94 ^i^ ± 0.04	14.18 ^l^ ± 0.01
VAR10	136.33 ^ab^ ± 2.49	74.47 ^cde^ ± 4.56	43.52 ^fgh^ ± 7.73	26.27 ^ab^ ± 3.27	32.27 ^def^ ± 5.50	3.74 ^bcd^ ± 0.71	5.68^def^ ± 0.40	0.0211 ^defg^ ± 0.0056	6.80 ^c^ ± 0.02	1.47 ^hi^ ± 0.01	4.63 ^a^ ± 0.05	12.65 ^o^ ± 0.02
VAR11	126.33 ^cdefg^ ± 3.30	75.87 ^cd^ ± 9.91	33.79 ^h^ ± 4.93	20.13 ^ef^ ± 2.98	25.47 ^gh^ ± 3.75	2.75 ^ef^ ± 0.52	3.78 ^gh^ ± 0.17	0.0258 ^bcd^ ± 0.0059	4.44 ^l^ ± 0.02	1.41 ^kl^ ± 0.01	3.15 ^h^ ± 0.01	17.25 ^d^ ± 0.02
VAR12	110.00 ^ij^ ± 7.48	36.33 ^k^ ± 2.85	85.55 ^a^ ± 3.21	24.20 ^bcd^ ± 1.07	31.00 ^defg^ ± 1.41	4.04 ^b^ ± 0.16	4.85 ^efg^ ± 0.14	0.0348 ^a^ ± 0.0011	5.32 ^g^ ± 0.02	1.50 ^gh^ ± 0.02	3.56 ^e^ ± 0.04	15.67 ^h^ ± 0.02
VAR13	129.00 ^bcdef^ ± 6.98	62.93 ^fgh^ ± 3.83	41.81 ^gh^ ± 4.93	22.27 ^cde^ ± 1.11	26.13 ^fgh^ ± 1.67	2.74 ^ef^ ± 0.19	3.11 ^h^ ± 0.09	0.0262 ^bcd^ ± 0.0005	5.43 ^f^ ± 0.03	1.52 ^fg^ ± 0.02	3.57 ^e^ ± 0.05	13.23 ^n^ ± 0.03
VAR14	132.67 ^abcd^ ± 1.89	55.17 ^ghij^ ± 6.58	57.81 ^cd^ ± 2.27	24.10 ^bcd^ ± 1.64	31.80 ^defg^ ± 3.15	2.91 ^def^ ± 0.17	6.28^cde^ ± 0.49	0.0175 ^fghi^ ± 0.0019	3.92 ^q^ ± 0.02	1.42 ^jk^ ± 0.02	2.76 ^k^ ± 0.05	14.45 ^j^ ± 0.02
VAR15	129.67 ^bcdef^ ± 6.85	57.27 ^fghi^ ± 4.66	52.49 ^defg^ ± 5.78	14.20 ^h^ ± 1.56	29.87 ^efg^ ± 2.19	2.48 ^f^ ± 0.12	9.66 ^a^ ± 1.15	0.0110 ^jk^ ± 0.0012	5.22 ^h^ ± 0.02	1.52 ^fg^ ± 0.02	3.43 ^f^ ± 0.03	14.25 ^k^ ± 0.01
VAR16	125.33 ^cdefg^ ± 0.47	83.47 ^bc^ ± 2.04	51.76 ^defg^ ± 3.30	17.37 ^fg^ ± 2.05	43.27 ^b^ ± 3.77	2.75 ^ef^ ± 0.23	10.31 ^a^ ± 0.27	0.0117 ^ijk^ ± 0.0005	6.29 ^d^ ± 0.02	1.44 ^ijk^ ± 0.03	4.36 ^b^ ± 0.09	16.72 ^e^ ± 0.02
VAR17	119.33 ^gh^ ± 0.47	90.60 ^ab^ ± 2.37	43.24 ^gh^ ± 1.85	14.67 ^gh^ ± 2.05	39.13 ^bc^ ± 0.66	3.72 ^bcd^ ± 0.14	9.66 ^a^ ± 1.44	0.0138 ^hijk^ ± 0.0017	6.85 ^b^ ± 0.02	1.72 ^c^ ± 0.01	3.99 ^c^ ± 0.04	18.45 ^b^ ± 0.02
VAR18	124.67 ^defg^ ± 1.25	67.37 ^def^ ± 2.21	54.66 ^def^ ± 1.70	13.73 ^h^ ± 1.52	36.80 ^cd^ ± 1.02	2.79 ^ef^ ± 0.26	10.68 ^a^ ± 0.54	0.0116 ^ijk^ ± 0.0014	8.21 ^a^ ± 0.02	1.85 ^a^ ± 0.01	4.44 ^b^ ± 0.02	16.30 ^g^ ± 0.05
VAR19	125.67 ^cdefg^ ± 0.47	99.97 ^a^ ± 8.87	41.41 ^gh^ ± 0.89	25.00 ^abc^ ± 0.73	41.40 ^bc^ ± 3.90	3.43 ^bcde^ ± 0.16	7.88 ^b^ ± 1.13	0.0161 ^ghij^ ± 0.0022	5.53 ^e^ ± 0.01	1.68 ^d^ ± 0.03	3.29 ^g^ ± 0.05	17.97 ^c^ ± 0.06
VAR20	121.00 ^efgh^ ± 1.73	76.43 ^cd^ ± 8.38	42.28 ^gh^ ± 4.54	21.67 ^cde^ ± 1.89	32.00 ^defg^ ± 2.20	2.73 ^ef^ ± 0.09	9.78 ^a^ ± 0.33	0.0127 ^ijk^ ± 0.0006	4.54 ^k^ ± 0.01	1.45 ^ij^ ± 0.03	3.13^h^ ± 0.05	11.92 ^q^ ± 0.02
VAR21	130.67 ^abcd^ ± 2.41	51.50 ^hij^ ± 1.35	43.97 ^efgh^ ± 1.98	23.10 ^bcde^ ± 0.69	22.67 ^h^ ± 1.56	1.63 ^g^ ± 0.20	7.53 ^bc^ ± 0.16	0.0078 ^k^ ± 0.0011	4.34 ^m^ ± 0.02	1.34 ^m^ ± 0.02	3.25 ^g^ ± 0.05	13.23 ^n^ ± 0.02
CV	6.96%	27.11%	38.92%	17.10%	18.59%	23.85%	18.76%	24.67%	20.91%	10.39%	20.59%	16.48%

Trait abbreviations are defined in Table 1.

**Table 3:** Analysis of variance (ANOVA) and genetic variability parameters for agro-morphological, yield, and grain-quality traits of 21 rice genotypes.

Trait	Block (df = 2)	MS(G)	MS(E)	GV	PV	GCV (%)	PCV (%)	H^2^ (%)	GA	GAM (%)
DF50	208.21	239.45 *	41.24	66.07	107.31	7.80	9.94	0.62	13.14	12.61
PH	25.84	690.71 ***	43.58	215.71	259.29	11.32	12.41	0.83	27.60	21.27
PL	3.46	28.18 ***	3.83	8.12	11.94	10.58	12.83	0.68	4.84	17.97
FLL	0.68	170.52 ***	11.55	52.99	64.54	18.33	20.22	0.82	13.59	34.21
FLW	0.02	0.16 ***	0.01	0.05	0.06	19.44	21.72	0.80	0.41	35.85
FLA	7.53	281.18 ***	22.68	86.16	108.85	27.38	30.78	0.79	17.01	50.19
FL:W	16.79	197.09 ***	22.45	58.21	80.66	21.05	24.78	0.72	13.35	36.83
NTT	0.67	15.17 ***	0.97	4.73	5.70	20.99	23.03	0.83	4.08	39.40
NPT	0.17	14.39 ***	0.93	4.49	5.41	20.42	22.43	0.83	3.97	38.28
NGPP	362.90	7855.60 ***	456.04	2466.51	2922.55	28.61	31.14	0.84	93.99	54.14
DM	3.64	225.68 ***	24.30	67.13	91.43	6.51	7.60	0.73	14.46	11.49
BM	114.61	890.13 ***	38.80	283.78	322.58	26.37	28.12	0.88	32.55	50.96
HI	46.40	405.21 ***	34.21	123.66	157.88	21.22	23.98	0.78	20.27	38.69
TW	28.69	43.45 ***	3.18	13.42	16.61	16.98	18.89	0.81	6.79	31.45
GYPP	21.77	138.14 ***	11.87	42.09	53.96	20.22	22.90	0.78	11.80	36.79
GYPPL	0.23	1.48 ***	0.19	0.43	0.62	20.59	24.79	0.69	1.12	35.23
GDPP	0.15	16.91 ***	0.70	5.41	6.10	35.06	37.25	0.89	4.51	67.97
KL	0.00	3.59 ***	0.00	1.20	1.20	21.19	21.20	1.00	2.25	43.65
KB	0.00	0.06 ***	0.00	0.02	0.02	8.95	9.05	0.98	0.28	18.25
L:B	0.00	1.00 ***	0.00	0.33	0.34	17.24	17.31	0.99	1.19	35.37
Amylose	0.00	14.49 ***	0.00	4.83	4.83	14.66	14.66	1.00	4.53	30.20

Significant at * *p* < 0.05, *** *p* < 0.001. Trait abbreviations are defined in Table 1.

**Table 4:** Phenotypic correlation matrix among agro-morphological, yield-related, and grain-quality traits of 21 rice genotypes.

Variables	DF50	PH	PL	FLL	FLW	FLA	FL:W	NTT	NPT	NGPP	DM	BM	HI	TW	GYPP	GYPPL	GDPP	EAGW	KL	KB	L:B	Amylose
**DF50**	1	−0.107	−0.210	−0.176	0.146	−0.040	−0.239	0.399 **	0.438 ***	0.454 ***	0.465 ***	0.555 ***	−0.417 ***	−0.191	0.347 **	−0.069	0.451 ***	−0.407 ***	0.365 **	0.295 *	0.276 *	0.111
**PH**	−0.107	1	0.521 ***	0.549 ***	0.123	0.433 ***	0.326 **	−0.505 ***	−0.559 ***	−0.448 ***	0.075	−0.169	0.055	0.411 ***	−0.297 *	0.204	−0.574 ***	0.487 ***	−0.137	−0.075	−0.094	−0.103
**PL**	−0.210	0.521 ***	1	0.325 **	0.199	0.334 **	0.109	−0.330 **	−0.367 **	−0.367 **	−0.001	−0.107	−0.174	0.309 *	−0.328 **	−0.007	−0.613 ***	0.258 *	−0.100	−0.164	−0.018	−0.146
**FLL**	−0.176	0.549 ***	0.325 **	1	0.130	0.739 ***	0.668 ***	−0.545 ***	−0.532 ***	−0.388 **	0.169	−0.288 *	0.020	0.393 **	−0.361 **	0.123	−0.431 ***	0.338 **	−0.279 *	−0.220	−0.188	−0.411 ***
**FLW**	0.146	0.123	0.199	0.130	1	0.756 ***	−0.616 ***	0.202	0.034	0.221	0.204	0.087	−0.211	0.171	−0.028	0.032	0.059	−0.180	−0.061	−0.011	−0.051	0.046
**FLA**	−0.040	0.433 ***	0.334 **	0.739 ***	0.756 ***	1	0.006	−0.250 *	−0.343 **	−0.113	0.243	−0.152	−0.108	0.363 **	−0.256 *	0.119	−0.246	0.117	−0.212	−0.149	−0.146	−0.236
**FL**:**W**	−0.239	0.326 **	0.109	0.668 ***	−0.616 ***	0.006	1	−0.470 ***	−0.368 **	−0.451 ***	−0.041	−0.309 *	0.143	0.184	−0.322 *	−0.032	−0.367 **	0.333 **	−0.224	−0.229	−0.137	−0.402 **
**NTT**	0.399 **	−0.505 ***	−0.330 **	−0.545 ***	0.202	−0.250 *	−0.470 ***	1	0.869 ***	0.584 ***	0.099	0.419 ***	−0.334 **	−0.358 **	0.268 *	−0.469 ***	0.610 ***	−0.715 ***	0.234	−0.003	0.265 *	0.141
**NPT**	0.438 ***	−0.559 ***	−0.367 **	−0.532 ***	0.034	−0.343 **	−0.368 **	0.869 ***	1	0.535 ***	0.189	0.395 **	−0.287 *	−0.359 **	0.314 *	−0.546 ***	0.605 ***	−0.737 ***	0.285 *	0.008	0.324 **	0.105
**NGPP**	0.454 ***	−0.448 ***	−0.367 **	−0.388 **	0.221	−0.113	−0.451 ***	0.584 ***	0.535 ***	1	0.162	0.459 ***	−0.154	−0.474 ***	0.499 ***	0.005	0.948 ***	−0.795 ***	0.526 ***	0.338 **	0.448 ***	0.303 *
**DM**	0.465 ***	0.075	−0.001	0.169	0.204	0.243	−0.041	0.099	0.189	0.162	1	0.241	−0.290 *	0.199	0.188	0.032	0.120	−0.197	−0.037	−0.055	0.003	−0.142
**BM**	0.555 ***	−0.169	−0.107	−0.288 *	0.087	−0.152	−0.309 *	0.419 ***	0.395 **	0.459 ***	0.241	1	−0.637 ***	−0.209	0.678 ***	0.213	0.419 ***	−0.272 *	0.392 **	0.312 *	0.321 *	0.382 **
**HI**	−0.417 ***	0.055	−0.174	0.020	−0.211	−0.108	0.143	−0.334 **	−0.287 *	−0.154	−0.290 *	−0.637 ***	1	0.160	0.077	0.356 **	−0.083	0.369 **	−0.022	0.157	−0.099	0.077
**TW**	−0.191	0.411 ***	0.309 *	0.393 **	0.171	0.363 **	0.184	−0.358 **	−0.359 **	−0.474 ***	0.199	−0.209	0.160	1	−0.139	0.215	−0.532 ***	0.433 ***	−0.420 ***	−0.360 **	−0.278 *	−0.395 **
**GYPP**	0.347 **	−0.297 *	−0.328 **	−0.361 **	−0.028	−0.256 *	−0.322 *	0.268 *	0.314 *	0.499 ***	0.188	0.678 ***	0.077	−0.139	1	0.605 ***	0.521 ***	−0.078	0.452 ***	0.537 ***	0.288 *	0.496 ***
**GYPPL**	−0.069	0.204	−0.007	0.123	0.032	0.119	−0.032	−0.469 ***	−0.546 ***	0.005	0.032	0.213	0.356 **	0.215	0.605 ***	1	−0.033	0.550 ***	0.154	0.420 ***	0.002	0.308 *
**GDPP**	0.451 ***	−0.574 ***	−0.613 ***	−0.431 ***	0.059	−0.246	−0.367 **	0.610 ***	0.605 ***	0.948 ***	0.120	0.419 ***	−0.083	−0.532 ***	0.521 ***	−0.033	1	−0.763 ***	0.503 ***	0.344 **	0.412 ***	0.277 *
**EAGW**	−0.407 ***	0.487 ***	0.258 *	0.338 **	−0.180	0.117	0.333 **	−0.715 ***	−0.737 ***	−0.795 ***	−0.197	−0.272 *	0.369 **	0.433 ***	−0.078	0.550 ***	−0.763 ***	1	−0.267 *	−0.008	−0.294 *	−0.047
**KL**	0.365 **	−0.137	−0.100	−0.279 *	−0.061	−0.212	−0.224	0.234	0.285 *	0.526 ***	−0.037	0.392 **	−0.022	−0.420 ***	0.452 ***	0.154	0.503 ***	−0.267 *	1	0.568 ***	0.891 ***	0.298 *
**KB**	0.295 *	−0.075	−0.164	−0.220	−0.011	−0.149	−0.229	−0.003	0.008	0.338 **	−0.055	0.312 *	0.157	−0.360 **	0.537 ***	0.420 ***	0.344 **	−0.008	0.568 ***	1	0.140	0.621 ***
**L:B**	0.276 *	−0.094	−0.018	−0.188	−0.051	−0.146	−0.137	0.265 *	0.324 **	0.448 ***	0.003	0.321 *	−0.099	−0.278 *	0.288 *	0.002	0.412 ***	−0.294 *	0.891 ***	0.140	1	0.033
**Amylose**	0.111	−0.103	−0.146	−0.411 ***	0.046	−0.236	−0.402 **	0.141	0.105	0.303 *	−0.142	0.382 **	0.077	−0.395 **	0.496 ***	0.308 *	0.277 *	−0.047	0.298 *	0.621 ***	0.033	1

*, **, *** indicate significance at *p* < 0.05, *p* < 0.01, and *p* < 0.001, respectively. Trait abbreviations are defined in Table 1.

**Table 5:** Genotypic correlation matrix among agro-morphological, yield-related, and grain-quality traits of 21 rice genotypes.

Variables	DF50	PH	PL	FLL	FLW	FLA	FL:W	NTT	NPT	NGPP	DM	BM	HI	TW	GYPP	GYPPL	GDPP	EAGW	KL	KB	L:B	Amylose
**DF50**	1	−0.109	−0.22	−0.221	0.183	−0.043	−0.32	0.528	0.561	0.659	0.513	0.79	−0.719	−0.358	0.474	−0.108	0.614	−0.617	0.48	0.358	0.383	0.145
**PH**	−0.109	1	0.695	0.677	0.136	0.531	0.451	−0.6	−0.681	−0.531	0.15	−0.169	0.027	0.545	−0.34	0.311	−0.663	0.635	−0.149	−0.078	−0.103	−0.114
**PL**	−0.22	0.695	1	0.503	0.269	0.492	0.228	−0.47	−0.505	−0.533	0.057	−0.098	−0.321	0.412	−0.483	−0.009	−0.741	0.42	−0.122	−0.18	−0.037	−0.177
**FLL**	−0.221	0.677	0.503	1	0.139	0.762	0.701	−0.661	−0.673	−0.407	0.127	−0.391	0.03	0.524	−0.496	0.158	−0.467	0.365	−0.31	−0.24	−0.213	−0.454
**FLW**	0.183	0.136	0.269	0.139	1	0.742	−0.584	0.223	0.051	0.288	0.18	0.09	−0.21	0.188	−0.028	0.008	0.084	−0.27	−0.068	−0.014	−0.058	0.051
**FLA**	−0.043	0.531	0.492	0.762	0.742	1	0.082	−0.322	−0.43	−0.085	0.207	−0.231	−0.085	0.465	−0.354	0.121	−0.255	0.072	−0.24	−0.169	−0.168	−0.265
**FL:W**	−0.32	0.451	0.228	0.701	−0.584	0.082	1	−0.604	−0.524	−0.555	−0.04	−0.417	0.132	0.297	−0.475	−0.007	−0.448	0.447	−0.267	−0.263	−0.169	−0.474
**NTT**	0.528	−0.6	−0.47	−0.661	0.223	−0.322	−0.604	1	0.964	0.722	0.146	0.481	−0.43	−0.46	0.301	−0.572	0.744	−0.875	0.26	−0.001	0.292	0.154
**NPT**	0.561	−0.681	−0.505	−0.673	0.051	−0.43	−0.524	0.964	1	0.662	0.212	0.466	−0.389	−0.46	0.367	−0.554	0.721	−0.836	0.314	0.011	0.356	0.114
**NGPP**	0.659	−0.531	−0.533	−0.407	0.288	−0.085	−0.555	0.722	0.662	1	0.235	0.594	−0.254	−0.52	0.622	−0.017	0.955	−0.852	0.573	0.376	0.487	0.332
**DM**	0.513	0.15	0.057	0.127	0.18	0.207	−0.04	0.146	0.212	0.235	1	0.287	−0.422	0.22	0.174	−0.026	0.157	−0.347	−0.046	−0.064	0.001	−0.164
**BM**	0.79	−0.169	−0.098	−0.391	0.09	−0.231	−0.417	0.481	0.466	0.594	0.287	1	−0.67	−0.269	0.734	0.185	0.515	−0.427	0.42	0.327	0.351	0.409
**HI**	−0.719	0.027	−0.321	0.03	−0.21	−0.085	0.132	−0.43	−0.389	−0.254	−0.422	−0.67	1	0.167	−0.033	0.385	−0.137	0.463	−0.026	0.187	−0.118	0.088
**TW**	−0.358	0.545	0.412	0.524	0.188	0.465	0.297	−0.46	−0.46	−0.52	0.22	−0.269	0.167	1	−0.265	0.215	−0.59	0.448	−0.482	−0.445	−0.305	−0.452
**GYPP**	0.474	−0.34	−0.483	−0.496	−0.028	−0.354	−0.475	0.301	0.367	0.622	0.174	0.734	−0.033	−0.265	1	0.56	0.635	−0.257	0.514	0.607	0.331	0.564
**GYPPL**	−0.108	0.311	−0.009	0.158	0.008	0.121	−0.007	−0.572	−0.554	−0.017	−0.026	0.185	0.385	0.215	0.56	1	−0.062	0.507	0.183	0.499	0.005	0.374
**GDPP**	0.614	−0.663	−0.741	−0.467	0.084	−0.255	−0.448	0.744	0.721	0.955	0.157	0.515	−0.137	−0.59	0.635	−0.062	1	−0.826	0.534	0.367	0.439	0.296
**EAGW**	−0.617	0.635	0.42	0.365	−0.27	0.072	0.447	−0.875	−0.836	−0.852	−0.347	−0.427	0.463	0.448	−0.257	0.507	−0.826	1	−0.303	−0.021	−0.329	−0.054
**KL**	0.48	−0.149	−0.122	−0.31	−0.068	−0.24	−0.267	0.26	0.314	0.573	−0.046	0.42	−0.026	−0.482	0.514	0.183	0.534	−0.303	1	0.575	0.894	0.298
**KB**	0.358	−0.078	−0.18	−0.24	−0.014	−0.169	−0.263	−0.001	0.011	0.376	−0.064	0.327	0.187	−0.445	0.607	0.499	0.367	−0.021	0.575	1	0.156	0.629
**L:B**	0.383	−0.103	−0.037	−0.213	−0.058	−0.168	−0.169	0.292	0.356	0.487	0.001	0.351	−0.118	−0.305	0.331	0.005	0.439	−0.329	0.894	0.156	1	0.033
**Amylose**	0.145	−0.114	−0.177	−0.454	0.051	−0.265	−0.474	0.154	0.114	0.332	−0.164	0.409	0.088	−0.452	0.564	0.374	0.296	−0.054	0.298	0.629	0.033	1

Trait abbreviations are defined in Table 1.

**Table 6:** Path coefficient analysis showing direct (diagonal) and indirect effects (off-diagonal) of agro-morphological and grain traits on grain yield per plant in 21 rice genotypes.

Traits	PH	PL	FLL	NTT	NPT	NGPP	BM	HI	TW	KL	KB	Amylose	Total_Correlation
**PH**	−0.2417	−0.00201	0.051637	0.107771	−0.13261	−0.11651	−0.12936	0.078427	0.097614	0.012036	−0.0308	−0.00419	−0.30969
**PL**	−0.13104	−0.0037	0.030447	0.064769	−0.08139	−0.09067	−0.06884	−0.06535	0.072469	0.007012	−0.05268	−0.00535	−0.32433
**FLL**	−0.13393	−0.00121	0.093187	0.110758	−0.12102	−0.09734	−0.20123	−0.0347	0.092119	0.021666	−0.07453	−0.01549	−0.36171
**NTT**	0.129735	0.001195	−0.0514	−0.20078	0.195387	0.145153	0.284895	−0.13955	−0.08445	−0.01705	−0.00579	0.005108	0.262444
**NPT**	0.142181	0.001337	−0.05003	−0.17403	0.225428	0.132865	0.268626	−0.13292	−0.08456	−0.02112	−0.00134	0.003749	0.31019
**NGPP**	0.112651	0.001344	−0.03629	−0.11659	0.119819	0.249974	0.315381	−0.12068	−0.11129	−0.04014	0.111772	0.011274	0.497221
**BM**	0.04511	0.000368	−0.02705	−0.08253	0.087367	0.113742	0.69312	−0.19121	−0.04903	−0.02955	0.102441	0.014264	0.677038
**HI**	−0.04685	0.000598	−0.00799	0.069248	−0.07405	−0.07455	−0.32753	0.404629	0.039486	0.000313	0.00353	0.003826	−0.00935
**TW**	−0.1007	−0.00115	0.03664	0.072375	−0.08137	−0.11875	−0.14506	0.068194	0.234286	0.03243	−0.12112	−0.01486	−0.13907
**KL**	0.037889	0.000338	−0.0263	−0.04459	0.062021	0.13069	0.266774	−0.00165	−0.09896	−0.07678	0.188994	0.011084	0.449523
**KB**	0.022166	0.000581	−0.02068	0.00346	−0.0009	0.083194	0.211421	0.004253	−0.08449	−0.04321	0.335841	0.023291	0.534929
**Amylose**	0.026981	0.000527	−0.03842	−0.02731	0.022504	0.075032	0.263223	0.041216	−0.09268	−0.02266	0.20825	0.03756	0.494234

Trait abbreviations are defined in Table 1.

**Table 7:** Multiple linear regression models identifying key agronomic and grain-quality traits associated with grain number per panicle (NGPP) and amylose content in 21 rice genotypes.

Dependent Variable	Predictor Trait	Standardized Coefficient (β)	*p*-Value	Significance
**Yield (NGPP)**	DF50	0.299	0.008	**
	PH	−0.275	0.015	*
	NTT	0.440	0.018	*
	NPT	−0.140	0.496	ns
	DM	0.031	0.772	ns
**Quality (Amylose)**	KL	−2.720	0.023	*
	KB	1.751	0.001	***
	LB_Ratio	2.111	0.033	*
	TW	−0.316	0.003	

*, **, *** indicate significance at *p* < 0.05, *p* < 0.01, and *p* < 0.001, respectively.

**Table 8:** Distribution of 21 rice genotypes among hierarchical clusters based on phenotypic similarity.

Cluster	No. of Genotypes	Genotypes
Cluster I	13	VAR4, VAR16, VAR3, VAR5, VAR21, VAR6, VAR15, VAR19, VAR2, VAR20, VAR12, VAR17, VAR18
Cluster II	3	VAR1, VAR9, VAR11
Cluster III	1	VAR14
Cluster IV	4	VAR7, VAR13, VAR8, VAR10

**Table 9:** Ranking of 21 rice varieties/genotypes based on grain yield per plant (GYPP) along with their amylose content and length-to-breadth (L:B) ratio, and overall performance status.

Rank	Variety	Grain Yield Per Plant (GYPP)	Amylose Content (%)	L:B Ratio	Performance Status
1	VAR16	43.27	16.72	4.36	Superior
2	VAR12	31.00	15.67	3.56	Superior
3	VAR1	50.47	18.87	2.89	Superior
4	VAR17	39.13	18.45	3.99	High Performing
5	VAR18	36.80	16.3	4.44	High Performing
6	VAR19	41.40	17.97	3.29	Intermediate
7	VAR10	32.27	12.65	4.63	Intermediate
8	VAR4	25.87	15.24	3.12	Intermediate
9	VAR15	29.87	14.25	3.43	Intermediate
10	VAR2	29.6	16.26	2.87	Intermediate
11	VAR8	33.07	11.26	3.71	Intermediate
12	VAR6	26.40	16.62	2.79	Low Performing
13	VAR20	32.00	11.92	3.13	Low Performing
14	VAR14	31.80	14.45	2.76	Low Performing
15	VAR9	27.13	14.18	2.94	Low Performing
16	VAR3	31.87	14.23	2.94	Low Performing
17	VAR11	25.47	17.25	3.15	Low Performing
18	VAR13	26.13	13.23	3.57	Low Performing
19	VAR7	28.27	13.73	2.78	Low Performing
20	VAR21	22.67	13.23	3.25	Poor
21	VAR5	29.27	12.26	2.78	Poor

## Data Availability

The raw data supporting the conclusions of this article will be made available by the authors on request.

## Supplementary Materials

The following supporting information can be downloaded at: https://www.mdpi.com/article/10.3390/plants15142134/s1, Table S1: Description of the experimental rice genotypes, including ecology, material type, origin, and maturity duration.

## References

[1] Timmer C.P. (2014). Food security in Asia and the Pacific: The rapidly changing role of rice. Asia Pac. Policy Stud..

[2] Muthayya S., Sugimoto J.D., Montgomery S., Maberly G.F. (2014). An overview of global rice production, supply, trade, and consumption. Ann. N. Y. Acad. Sci..

[3] Bandumula N. (2018). Rice production in Asia: Key to global food security. Proc. Natl. Acad. Sci. India Sect. B Biol. Sci..

[4] Khush G.S. (2013). Strategies for increasing the yield potential of cereals: Case of rice as an example. Plant Breed..

[5] Siddiq E., Vemireddy L.R. (2021). Advances in genetics and breeding of rice: An overview. Rice Improvement: Physiological, Molecular Breeding and Genetic Perspectives.

[6] Ray D.K., Mueller N.D., West P.C., Foley J.A. (2013). Yield trends are insufficient to double global crop production by 2050. PLoS ONE.

[7] Peng S., Huang J., Sheehy J.E., Laza R.C., Visperas R.M., Zhong X., Centeno G.S., Khush G.S., Cassman K.G. (2004). Rice yields decline with higher night temperature from global warming. Proc. Natl. Acad. Sci. USA.

[8] Welch J.R., Vincent J.R., Auffhammer M., Moya P.F., Dobermann A., Dawe D. (2010). Rice yields in tropical/subtropical Asia exhibit large but opposing sensitivities to minimum and maximum temperatures. Proc. Natl. Acad. Sci. USA.

[9] Hill W.G., Mackay T.F. (2004). DS Falconer and Introduction to quantitative genetics. Genetics.

[10] Hair J., Sabol M.A. (2025). Overview of Multivariate Data Analysis. International Encyclopedia of Statistical Science.

[11] Goldschmidt R.B. (2022). Theoretical Genetics.

[12] Jolliffe I. (2025). Principal Component Analysis. International Encyclopedia of Statistical Science.

[13] Wright S. (1921). Correlation and causation. J. Agric. Res..

[14] Jolliffe I.T., Cadima J. (2016). Principal component analysis: A review and recent developments. Philos. Trans. R. Soc. A Math. Phys. Eng. Sci..

[15] Dewey D.R., Lu K. (1959). A correlation and path-coefficient analysis of components of crested wheatgrass seed production. Agron. J..

[16] Tu Anh T.T., Tu Anh T.T., Khanh T.D., Dat T.D., Xuan T.D. (2018). Identification of phenotypic variation and genetic diversity in rice (*Oryza sativa* L.) mutants. Agriculture.

[17] Ogunbayo S., Sie M., Ojo D.K., Sanni K.A., Akinwale M.G., Toulou B., Shittu A., Idehen E.O., Popoola A.R., Daniel I.O. (2014). Genetic variation and heritability of yield and related traits in promising rice genotypes (*Oryza sativa* L.). J. Plant Breed. Crop Sci..

[18] Pandey P., Anurag P.J., Tiwari D., Yadav S., Kumar B. (2009). Genetic variability, diversity and association of quantitative traits with grain yield in rice (*Oryza sativa* L.). J. Bio-Sci..

[19] Asante M.D., Adjah K.L., Annan-Afful E. (2019). Assessment of genetic diversity for grain yield and yield component traits in some genotypes of rice (*Oryza sativa* L.). J. Crop Sci. Biotechnol..

[20] Tuhina-Khatun M., Hanafi M.M., Rafii Yusop M., Wong M., Salleh F.M., Ferdous J. (2015). Genetic variation, heritability, and diversity analysis of upland rice (*Oryza sativa* L.) genotypes based on quantitative traits. BioMed Res. Int..

[21] Salem K., Saleh M.M., Aldahak L., Elabd A.B. (2021). Assessment phenotypic diversity of rice (*Oryza sativa* L.) genotypes by multivariate analysis. J. Arid. Agric..

[22] Debsharma S.K., Syed M.A., Ali M.H., Maniruzzaman S., Roy P.R., Brestic M., Gaber A., Hossain A. (2022). Harnessing on genetic variability and diversity of rice (*Oryza sativa* L.) genotypes based on quantitative and qualitative traits for desirable crossing materials. Genes.

[23] Islam M.Z., Chakrabarty T., Akter N., Khalequzzaman M., Prince M.F.R.K., Pittendrigh B.R., Tomita M., Ali M.P. (2025). Genetic variability, correlation and path coefficient analysis of phenotypic traits and genetic diversity of Aman rice landraces (*Oryza sativa* L.). Sci. Rep..

[24] Nath S., Kole P. (2021). Genetic variability and yield analysis in rice. Electron. J. Plant Breed..

[25] Bagheri N., Babaeian-Jelodar N., Pasha A. (2011). Path coefficient analysis for yield and yield components in diverse rice (*Oryza sativa* L.) genotypes. Biharean Biol..

[26] Idris A., Justin F., Dagash Y., Abuali A. (2012). Genetic variability and inter relationship between yield and yield components in some rice genotypes. Am. J. Exp. Agric..

[27] Oladosu Y., Rafii M., Magaji U., Abdullah N., Miah G., Chukwu S.C., Hussin G., Ramli A., Kareem I. (2018). Genotypic and phenotypic relationship among yield components in rice under tropical conditions. BioMed Res. Int..

[28] Kiani G. (2012). Character association and path coefficient analysis of yield components in rice varieties. Res. Crops.

[29] Sudeepthi K., Srinivas T.V.S.R., Kumar B.R., Jyothula D.P.B., Umar S.N. (2020). Assessment of genetic variability, character association and path analysis for yield and yield component traits in rice (*Oryza sativa* L.). Electron. J. Plant Breed..

[30] Prakash S., Reddy S.S., Chaudhary S., Vimal S.C., Kumar A. (2024). Multivariate analysis in rice (*Oryza sativa* L.) germplasms for yield attributing traits. Plant Sci. Today.

[31] Krishna K., Chandra Mohan Y., Krishna L., Parimala G., Jagadeeshwar R. (2022). Multivariate analysis based prediction of phenotypic diversity associated with yield and yield component traits in germplasm lines of rice (*Oryza sativa* L.). Electron. J. Plant Breed..

[32] Maji A., Shaibu A. (2012). Application of principal component analysis for rice germplasm characterization and evaluation. J. Plant Breed. Crop Sci..

[33] Sanni K., Fawole I., Ogunbayo S., Tia D., Somado E., Futakuchi K., Sié M., Nwilene F.E., Guei R.G. (2012). Multivariate analysis of diversity of landrace rice germplasm. Crop Sci..

[34] Shoba D., Vijayan R., Robin S., Manivannan N., Iyanar K., Arunachalam P., Geetha S. (2019). Assessment of genetic diversity in aromatic rice (*Oryza sativa* L.) germplasm using PCA and cluster analysis. Electron. J. Plant Breed..

[35] Ovung C.Y., Lal G., Rai P.K. (2012). Studies on genetic diversity in rice (*Oryza sativa* L.). J. Agric. Technol..

[36] Alia, Khalil H.I., Shah S.M.A., Durrishahwar M.N. (2021). 03. Grouping rice genotypes in different clusters based on their similarity index. Pure Appl. Biol..

[37] Alia, Khalil H.I., Shah S.M.A., Durrishahwar M.N. (2016). Grouping rice genotypes in different clusters based on their similarity index. Pure Appl. Biol..

[38] Al Galib M.A., Farzana S., Chakrobarty T., Islam M.Z., Shirazy B.J., Rahman M.A., Imran S., Tahjib-Ul-Arif M., Rhaman M.S. (2025). Multivariate analysis for identifying high-yielding rice cultivars based on seed yield and morphological traits. Discov. Plants.

[39] Hajjar M.J., Ahmed N., Alhudaib K.A., Ullah H. (2023). Integrated insect pest management techniques for rice. Sustainability.

[40] Guttikonda H., Chandu G., Munnam S.B., Beerelli K., Balakrishnan D., Ragimasalawada M., Sarla N. (2022). Oryza nivara allele of a major effect QTL *qFLA1.1* increases flag leaf area in rice. Res. Sq..

[41] Ashfaq M., Khan A.S., Ullah Khan S.H., Ahmad R. (2012). Association of various morphological traits with yield and genetic divergence in rice (Oryza sativa). Int. J. Agric. Biol..

[42] Rao D.S., Subramanyam D., Suneetha K., Azam M., Babu V.R. (2014). Different methods of amylose estimation and their comparision. J. Res. ANGRAU.

