# Supplementary material for: Dissecting Yield Architecture and Trait Interactions in Rice Using Integrative Multivariate Selection Index and Phenotypic Similarity Analysis
Source: Plants (Basel). 2026 Jul 10;15(14):2134. doi: 10.3390/plants15142134 (PMC13414536; doi:10.3390/plants15142134)
Supplement: Supplementary file 1 [file plants-15-02134-s001.zip › plants-4399177-supplementary.pdf]

Supplemetray table

Table S1: Description of the experimental rice genotypes, including ecology, material type, origin, and maturity duration.

Variety	Ecology	Material Type	Origin	Duration
VAR1	Low land	Breeding line	Indica	139
VAR2	Low land	Breeding line	Indica	140
VAR3	Low land	Breeding line	Indica	132
VAR4	Low land	Breeding line	Indica	102
VAR5	Low land	Released Variety	Indica	136
VAR6	Low land	Released Variety	Indica	119
VAR7	Low land	Released Variety	Indica	120
VAR8	Low land	Released Variety	Indica	132
VAR9	Low land	Released Variety	Indica	120
VAR10	Low land	Released Variety	Indica	139
VAR11	Low land	Released Variety	Indica	127
VAR12	Low land	Released Variety	Indica	108
VAR13	Low land	Released Variety	Indica	120
VAR14	Low land	Released Variety	Indica	130
VAR15	Low land	Released Variety	Indica	135
VAR16	Low land	Released Variety	Indica	125
VAR17	Low land	Released Variety	Indica	120
VAR18	Low land	Released Variety	Indica	123
VAR19	Low land	Released Variety	Indica	126
VAR20	Low land	Released Variety	Indica	120
VAR21	Low land	Released Variety	Indica	128
